# FIMBRIN2 Regulates ABA‐Induced Stomatal Closure by Promoting Microfilament Bundling and Turnover in Arabidopsis Guard Cells

**DOI:** 10.1002/pld3.70162

**Published:** 2026-04-12

**Authors:** Zixuan Wang, Yuchong Han, Pan Wang, Pengfang Sun, Miao Hu, Rong Yu

**Affiliations:** ^1^ College of Life Sciences Capital Normal University Beijing China; ^2^ State Key Laboratory of Plant Environmental Resilience, College of Biological Sciences China Agricultural University Beijing China

**Keywords:** abiotic stress, abscisic acid, actin‐binding protein, *Arabidopsis thaliana*, drought resistance, FIMBRIN, microfilaments, stomatal closure

## Abstract

In terrestrial plants, drought stress activates abscisic acid (ABA) signaling in guard cells, prompting stomatal closure to reduce water loss. Stomatal closure is accompanied by reorganization of the microfilaments. However, the mechanism by which ABA signaling regulates microfilaments disassembly remains unclear, and the actin‐binding proteins (ABPs) involved in this process have yet to be fully identified. In this study, we demonstrated that FIMBRIN2 (FIM2), an actin‐bundling protein, has its expression in guard cells upregulated by ABA, and FIM2 is involved in ABA‐induced stomatal closure. The *fim2* mutant exhibits a drought‐sensitive phenotype and delayed stomatal closure. Further confocal microscopy observations confirmed that this delay results from impaired actin bundling and reduced actin turnover activity. These findings reveal FIM2's role in ABA‐induced stomatal closure and deepen our understanding of the functions of ABPs and the actin cytoskeleton in plant stress adaptation.

**Abbreviations**
ABA: abscisic acid
ABD: actin‐binding domain
ABPs: actin‐binding proteins
CH domain: calponin‐homology domain
FIM2: FIMBRIN2
FRAP: fluorescence recovery after photobleaching
GFP: green fluorescent protein
Jas: jasplakinolide
Lat B: latrunculin B

## 1 Introduction

Stomata are small pores located on the surfaces of leaves and stems in terrestrial plants, each composed of a pair of guard cells. Through these specialized structures, plants regulate gas exchange with the external environment (including water and carbon dioxide) to maintain homeostasis (Blatt 2000). Not surprisingly, stomata connect the global water and carbon cycles coordinately. Understanding how environmental factors influence stomatal movement and plant fitness is becoming increasingly crucial (Nguyen et al. 2023; Chua and Lau 2024). The regulation of stomatal behavior is achieved through changes in guard cell volume and shape, thereby modulating the stomatal opening and closing. This process is heavily controlled by a sophisticated signal transduction network, plants can convert perceived environmental cues such as drought, pathogen or heat into internal phytohormone signal. Abscisic acid (ABA) is a vital phytohormone that responds to drought stress and promotes stomatal closure to minimize unnecessary water loss (Munemasa et al. 2015; Kuromori et al. 2018; Bharath et al. 2021). During the ABA‐induced stomatal closing, a rapid, precise microfilaments reorganization is indispensable (Li et al. 2022), enabling the regulation of ion channels, membrane protein function, like aquaporin AtPIP2 and vacuole morphology, and vesicle trafficking in guard cells (Zhang and Fan 2009; MacRobbie and Kurup 2007; Cui et al. 2021; Akkerman et al. 2011; Zheng et al. 2019).

Actin array rearrangements in the process of stomatal movement are commonly categorized into three distinct types. Type I “radial arrays,” commonly seen in open stomata, microfilaments formed short bundles are distributed radially from stomatal pores. Type II “random meshwork,” which is considered as a transition state between Type I and Type III, microfilaments are randomly distributed and organized into mesh‐like networks. Type III “longitudinal arrays”, microfilaments formed long, longitudinally distributed bundles (Shimono et al. 2016; Isner et al. 2017). Increasing evidence points out that various actin‐binding proteins (ABPs) are excellent candidates for signal relay in actin remodeling during stomatal movement. For example, actin‐depolymerizing factor5 (ADF5) promotes stomatal closure by regulating actin cytoskeleton remodeling in response to ABA and drought stress, probably as a potential downstream target of ABF/AREB transcription factor DPBF3 (Qian and Zhang 2019). ADF4, another ADF, could be phosphorylated by casein kinase 1‐like protein 2 (CKL2), facilitating the reassembly of actin filaments in ABA‐induced stomatal closure (Zhao et al. 2016). A plant‐specific ABP (stomatal closure‐related actin‐binding protein1, SCAB1), which bundles and stabilizes actins, has been demonstrated to regulate the stability and rearrangement of F‐actin in guard cells during ABA‐induced stomatal closure and is involved in the phosphatidylinositol 3‐phosphate (PI3P) signaling pathway (Zhao et al. 2011; Yang et al. 2022). The paralogous Arabidopsis KASH proteins SINE1 and SINE2 have synergistic and antagonistic interactions with SCAB1 and actin‐related protein2/3 (ARP2/3) in actin dynamics during stomatal movement in response to ABA signaling (Moser et al. 2024). It is noteworthy that various ABPs work together or specifically to keep the fine tune of actin remodeling and thus stomatal movement in response to external stimuli (Wang and Mao 2019; Lian et al. 2021).

The fimbrins, also known as plastins, are unique among the actin‐crosslinking proteins, as they possess two tandem repeats of the actin‐binding domain (ABD) within a single polypeptide chain. Due to the close proximity of the ABDs, fimbrin directs the formation of tightly bundled F‐actin structures (Klein et al. 2004). Arabidopsis has five *FIMBRIN* genes, *FIMBRIN1(FIM1)‐FIM5*. It has been well documented that AtFIM1, AtFIM4, and AtFIM5 are all crucial for maintaining normal actin architecture in pollen tubes and play a role in pollen development (Kovar et al. 2000; Zhang et al. 2016; Su et al. 2017). The lily ( *Lilium longiflorum* ) homologue of AtFIM5, LI‐FIM1, has also been implicated in regulating the actin organization within the subapical region of the pollen tube (Su et al. 2012). Moreover, AtFIM4 and AtFIM5 regulate the protein abundance of the auxin synthesis enzyme YUC8 and modulate endogenous auxin levels in roots, consequently impact root development (Liu et al. 2024). How about AtFIM2? In 2004, microarray expression analyses of Arabidopsis guard cells revealed that compared with mesophyll cells, *FIMBRIN 2* (At5g48460) is one of the 64 transcripts preferentially expressed in guard cells, and a strong candidate for contributing to guard cell signal transduction (Leonhardt et al. 2004). However, the role and underlying mechanisms of FIM2‐microfilament modules in this process are not well understood.

In this study, we found that the expression of *FIM2* in guard cells is upregulated by ABA, and *Arabidopsis* FIM2 is involved in ABA‐induced stomatal movement. Mechanistically, we show that FIM2 positively regulates actin filament (F‐actin) disassembly/assembly dynamic instability and facilitates actin filaments bundling, which consequently results in F‐actin realignment during stomatal closure in response to drought stress. Our work thus provides valuable information about FIM2‐mediated actin behaviors in stomatal movement.

## 2 Results

### 2.1 *FIM2* Mutation Weakens Drought Tolerance in *Arabidopsis thaliana*

**FIGURE 1:**
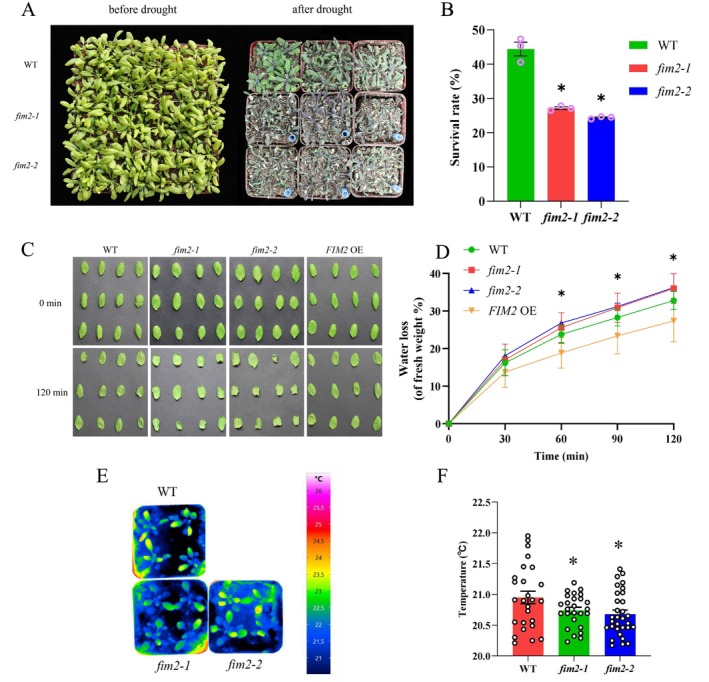
*fim2* mutants weakens drought tolerance in *Arabidopsis thaliana.* (A) Drought phenotype of different seedlings in soil. Fourteen‐day‐old seedlings were planted into soil drying by withholding water for 2 weeks. (B) The survival rate was calculated from biological independent experiments, mean ± SD, two‐tailed *t*‐test, *p < 0.05. (C) Fresh weights of detached leaves from different seedlings was measured every 30 min for a total of 2 h. Values from three biological independent experiments, each with 12 leaves. (D) Leave weights was measured at 30‐min intervals. Values represent the mean ± SD for three independent experiments, each with a minimum of 12 leaves, *p < 0.05 (two‐tailed *t*‐test). (E) Image captured by infrared camera (VarioCAM HD) of different seedlings in soil. (F) Leaf temperature was measured using infrared camera software IRBIS3 professional. The experiment was repeated three times independently as different biological replicates with a minimum of 20 leaves of each seedling. Different letters represent significant differences at *p < 0.05 (two‐tailed *t*‐test).

To investigate if FIM2 takes part in drought stress tolerance in *A. thaliana* , we obtained two parallel T‐DNA insertion mutants: *fim2‐1* and *fim2‐2*. We then tested their abilities in drought tolerance; 14‐day‐old soil‐grown seedlings were subjected to progressive drought stress conditions (Figure 1A). Following 14 days of water deprivation, *fim2* mutant lines exhibited obvious phenotypic symptoms including leaf desiccation and rolling compared to WT counterparts. WT plants demonstrated a significantly higher survival rate compared to *fim2* mutants (Figure 1B).

We then performed a water loss assay, which was described previously (Sedbrook et al. 2004). We detached 12 rosette leaves and measured their fresh weights every 30 min to determine the rate of water loss. Compared to the WT, the shrinkage of the *fim2* mutant leaves was more serious (Figure 1C). Also, it was found that the water loss rate of the two *fim2* mutants was significantly higher than that of the WT at 120 min (Figure 1D). In addition, *FIM2 OE* seedings showed a lower water loss rate, implying that FIM2 enhances drought tolerance in plants.

Furthermore, we conducted measurements of leaf temperature. The process of water loss through stomata removes heat, leading to a reduction in leaf temperature. As shown in Figure 1F, after treatment with ABA for 3 h, the leaf temperature of *fim2‐1* and *fim2‐2* was significantly lower than that of the WT. Taken together, these findings demonstrate that *fim2* mutants weaken drought tolerance in plants.

### 2.2 FIM2 Expression in Guard Cells Is Upregulated by ABA

**FIGURE 2:**
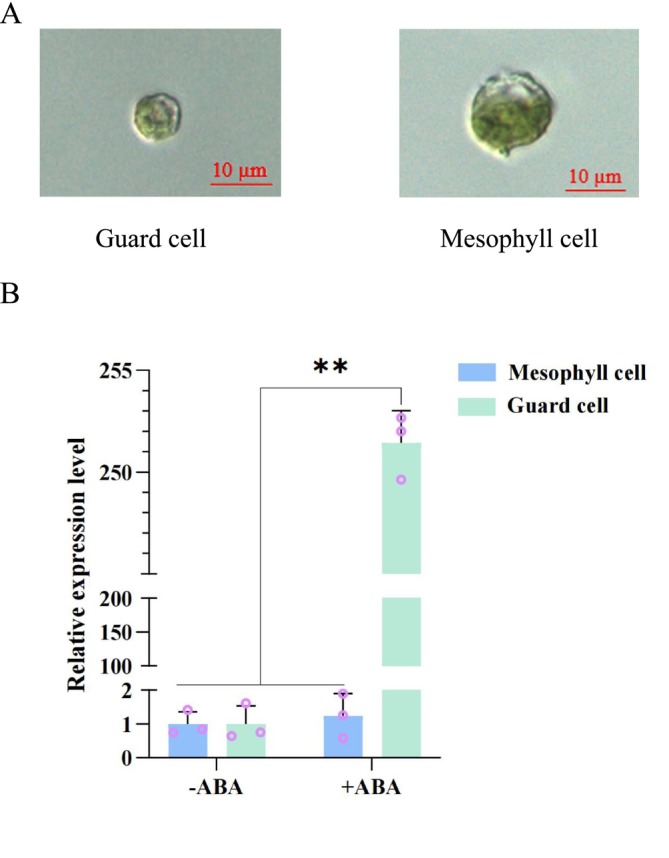
*FIM2*'s expression is upregulated by ABA. (A) Protoplasts of guard cells and mesophyll cells from *Arabidopsis thaliana* leave. (B) The relative expression of *FIM2* in mesophyll cell protoplasts and guard cell protoplasts with or without 10‐μM ABA treatment. The expression level was standardized with the reference *EF1‐α* gene, and three independent biological replicates were performed (mean ± SEM, two‐tailed *t*‐test, **p < 0.01).

ABA is a well‐known phytohormone triggered by drought stress. It promotes stomatal closure and reduces water loss (Kuromori et al. 2018). To investigate if the phenotype of *fim2* is related to ABA signaling, guard cell and mesophyll cell protoplasts were purified, respectively, as reported by Leonhardt et al. (2004) (Figure 2A). Total RNA was then extracted from the protoplasts and reverse‐transcribed into cDNA for qPCR analysis. The results showed that under ABA treatment, the expression level of FIM2 in Arabidopsis guard cells exhibited a sharp increase compared to that without ABA treatment (‐ABA), indicating an extremely sensitive response to ABA signals (Figure 2B). In contrast, no significant alterations in the expression level of FIM2 were observed in mesophyll cells, which stood in striking contrast to the pattern observed in guard cells. These findings suggest that upon induction by the ABA signaling molecule, the *FIM2* gene in guard cells undergoes a preferential response with upregulated expression.

### 2.3 FIM2 Participates in ABA‐Induced Stomatal Closure

**FIGURE 3:**
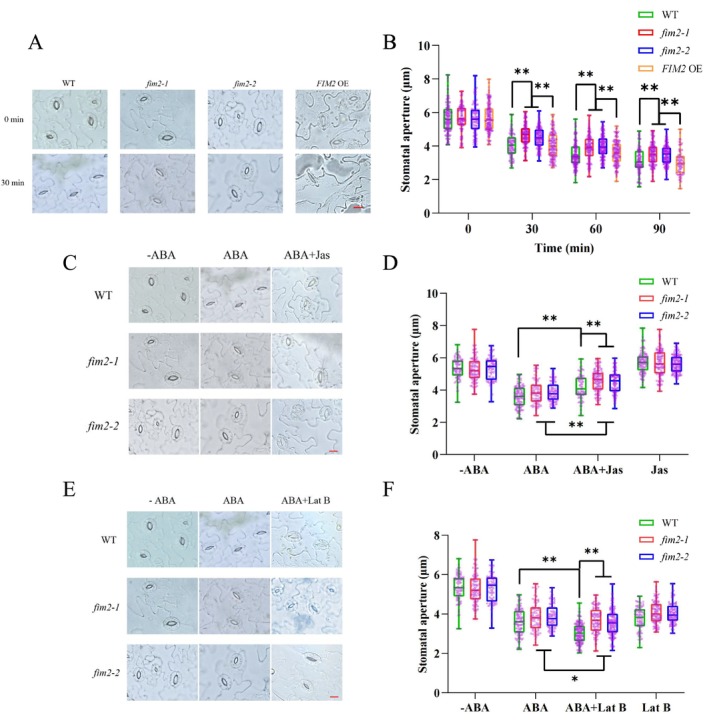
FIM2 participate in ABA‐induced stomatal closure. Measurement of stomatal aperture using different drug combinations. Rosette leaves 3–4 weeks old from different seedlings were detached and incubated in MES buffer for 3 h to ensure full stomatal opening. (A, B) Stomatal aperture of the *fim2* mutant, *FIM2 OE*, and WT over time under ABA induction. Pictures were captured by a 30‐min interval. (C, D) Stomatal aperture of WT, *fim2–1*, and *fim2–2* treat with ABA or ABA and Jas for 30 min. (E, F) Stomatal aperture of WT, *fim2–1*, and *fim2–2* treat with ABA or ABA and Lat B for 30 min. Both of the stomatal aperture experiments were repeated three times independently as different biological replicates with 70 stomata of each seedling. Different letters represent significant differences at *p < 0.05 or **p < 0.01 (mean ± SD, two‐tailed *t‐*test). Bar = 10 μm.

As a pivotal phytohormone in drought stress responses, ABA modulates drought response through stomatal closure to mitigate transpirational water loss. Stomata serve as the primary pathway for plant water loss (Lawson and Matthews 2020), and stomatal aperture reflects a plant's ability to tolerate drought stress. To determine whether the drought‐sensitive phenotype of the *fim2* mutant is caused by abnormal stomatal closure, we performed a stomatal aperture assay. Rosette leaves were detached from 3‐ to 4‐week‐old *A. thaliana* plants and placed in MES buffer, followed by illumination under a cool light irradiation for 3 h to facilitate full stomatal opening. Subsequently, the leaves were treated with ABA for 0–90 min, after which microscopic imaging and stomatal aperture measurement were performed at each time point. The results showed that the stomatal aperture of WT and FIM2 OE lines decreased significantly more than that of both *fim2* mutants (Figure 3B), indicating that FIM2 is a positive regulator of ABA‐induced stomatal closure.

In addition, ABA‐triggered stomatal closure is accompanied by microfilament reorganization (Li et al. 2022; Wang and Mao 2019). Therefore, we measured stomatal aperture under co‐treatment with ABA and the microfilament‐specific drugs Jasplakinolide (Jas) and Latrunculin B (Lat B) (Figure 3C,E). As shown in Figure 3D,F, under ABA treatment, *fim2* mutants were more sensitive to both microfilament‐specific drugs than the WT. Collectively, these results demonstrate that loss of FIM2 function may influence the microfilament dynamics of guard cells during ABA‐induced stomatal movement.

### 2.4 FIM2 Deficiency Disrupts ABA‐Responsive Microfilament Dynamics

**FIGURE 4:**
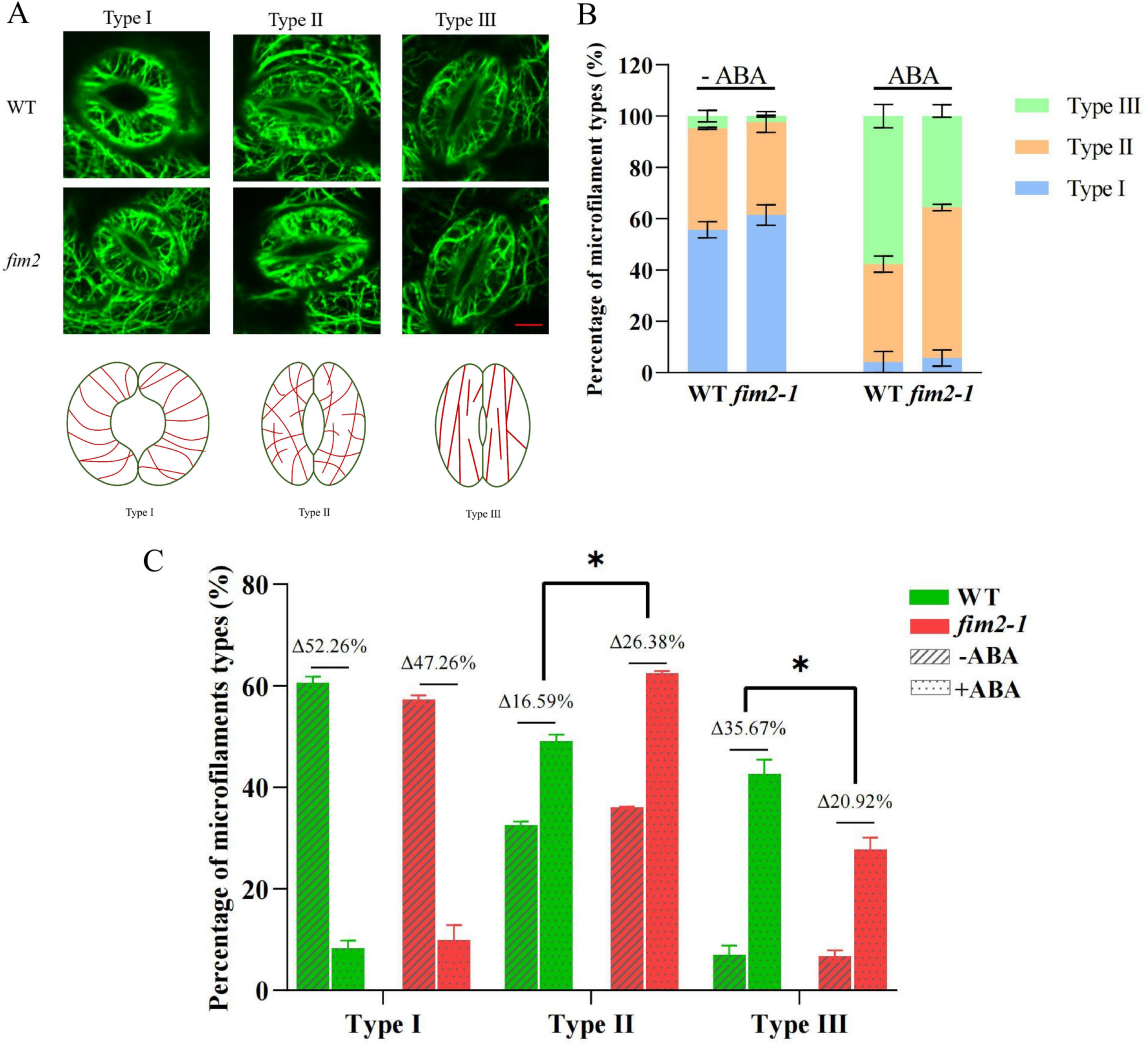
FIM2 deficiency interferes with normal microfilament organization pattern. (A) Confocal microscopy images and schematic illustration of microfilament arrangement in guard cells of WT and *fim2–1*. Bar = 2 μm. (B) Statistical data of WT and *fim2–1* on the proportion of microfilament arrangement. (C) Statistical data of WT and *fim2–1* on changes in the proportion of microfilament arrangement after ABA treatment. The data represent three biological independent experiments, with a total of 100 stomata measured (mean ± SD, two‐tailed *t*‐test, *p < 0.05).

During stomatal movement, guard cell microfilaments predominantly exhibit three arrangement patterns (Figure 4A): radial arrays (Type I), random meshwork (Type II), and fragmented/longitudinally oriented bundles (Type III). To further investigate how FIM2 deficiency impairs ABA‐induced microfilament reorganization, we generated a *fim2/GFP‐fABD2* line by crossing *fim2‐1* with *GFP‐fABD2* to conduct confocal microscopy observation.

**FIGURE 5:**
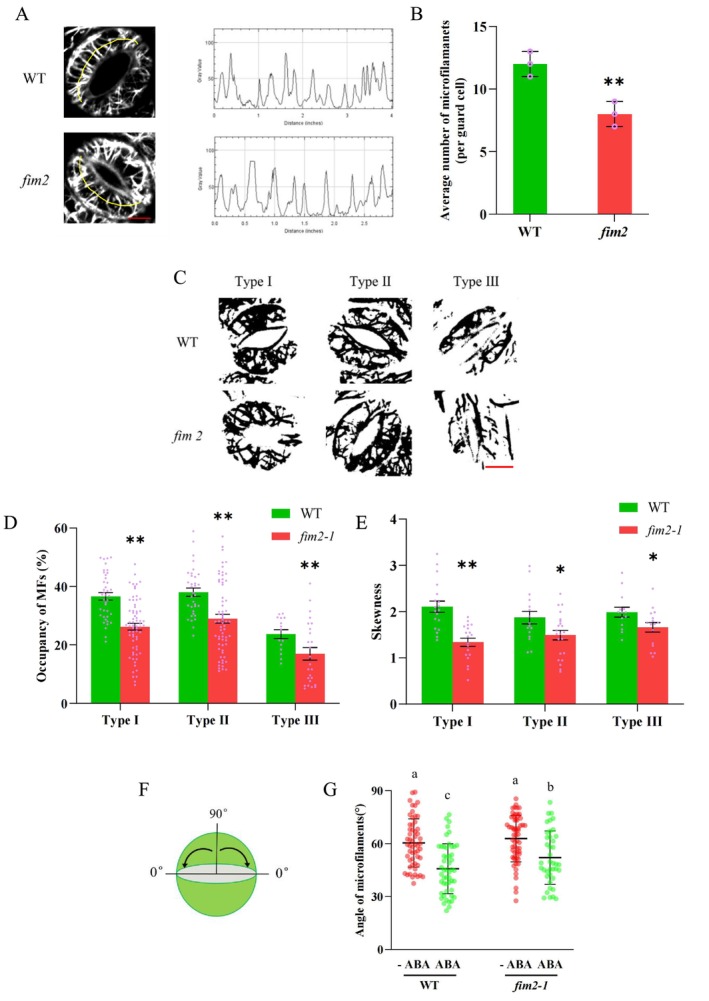
FIM2 deficiency disrupts ABA‐responsive microfilament dynamics. (A, B) The number of peaks with fluorescence intensity greater than 50 in radial microfilament bundles in guard cells (data from three independent experiments, *n* > 20 guard cells, mean ± SEM, two‐tailed *t*‐test, **p < 0.01). Bar = 5 μm. (C) Guard cells skeletonized using ImageJ software, Bar = 5 μm. (D) Occupancy of three types of microfilaments in guard cells of WT and *fim2–1* mutants (two‐tailed *t*‐test **p < 0.01). (E) Skewness (bundling ability) of three types of microfilaments in guard cells of WT and *fim2–1* mutants (two‐tailed *t*‐test *p < 0.05, **p < 0.01). (F) Schematic diagram of microfilament angle measurement. (G) Quantitative statistics of microfilament angles in guard cells (number of guard cells *n* ≥ 40, two‐tailed *t*‐test, *p < 0.05).

After 30 min of ABA treatment, compared to WT, the microfilament arrangement of *fim2* mutant mainly stays in Type II rather than transforms into Type III (Figure 4B,C). This phenomenon implies that *fim2* mutant lacks the ability to re‐bundle microfilaments. To further validate this hypothesis, we measured the number of visible microfilaments in guard cells. As shown in Figure 5B, the number of visible microfilaments in *fim2* guard cells was significantly reduced. Subsequently, we analyzed skeletonized microfilaments by Image J (Zou et al. 2021). *fim2* mutant was significantly lower in microfilaments occupancy (Figure 5D), skewness (Figure 5E), and angle (Figure 5G), indicating that under FIM2 deficiency, microfilament bundling process was hindered, microfilament bundle became thinner and spacier, and the microfilament organization failed to transform from Type II to Type III, thereby impairing ABA‐triggered stomatal closure.

### 2.5 *fim2* Mutant Exhibits Reduced Microfilaments Turnover Rate

**FIGURE 6:**
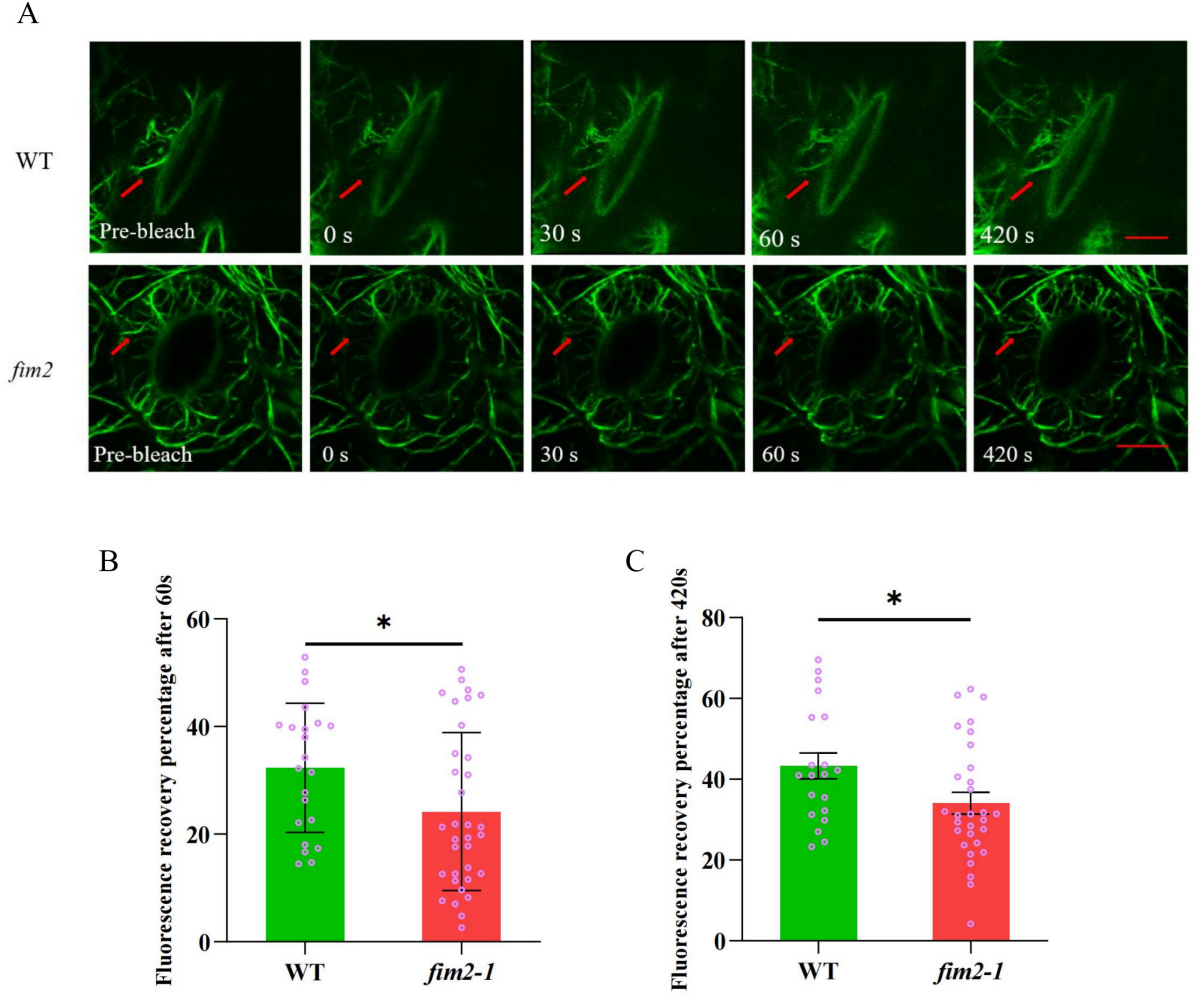
Fluorescence recovery after photobleaching (FRAP) assay was used to determine the effect of FIM2 on the dynamic turnover level of the microfilament cytoskeleton in guard cells. (A) Time‐lapse series of FRAP, Bar = 5 μm. (B) Fluorescence recovery ratio of microfilament bundles in the bleached region of guard cells at 60 and 420 s (data from three independent experiments, mean ± SEM, two‐tailed *t*‐test, *p < 0.05).

Based on previous findings, we performed FRAP assay to find out alterations in microfilament dynamics from a deeper level. As Figure 6B demonstrated, the fluorescence recovery ratio of *fim2* was significantly lower than WT, showing that *fim2*'s microfilament turnover rate was weakened.

### 2.6 FIM2 Affects ABA‐Induced ROS Dynamics in Guard Cells

Since stomatal closure is recognized as one of the most effective strategies to improve drought resistance, and H_2_O_2_ in guard cells is a key determinant of stomatal closure, primarily via the ABA‐dependent signaling pathway (Qi et al. 2018; Liu et al. 2022; Rodrigues and Shan 2022; Mittler et al. 2022; Yin et al. 2025; Liu and Tominaga 2025). H_2_O_2_ accumulation in guard cells is indispensable for ABA‐induced actin dynamics during stomatal closure (Li et al. 2014). Therefore, we performed a series of assays to investigate whether FIM2 participates in the ABA–H_2_O_2_ signaling cascade.

**FIGURE 7:**
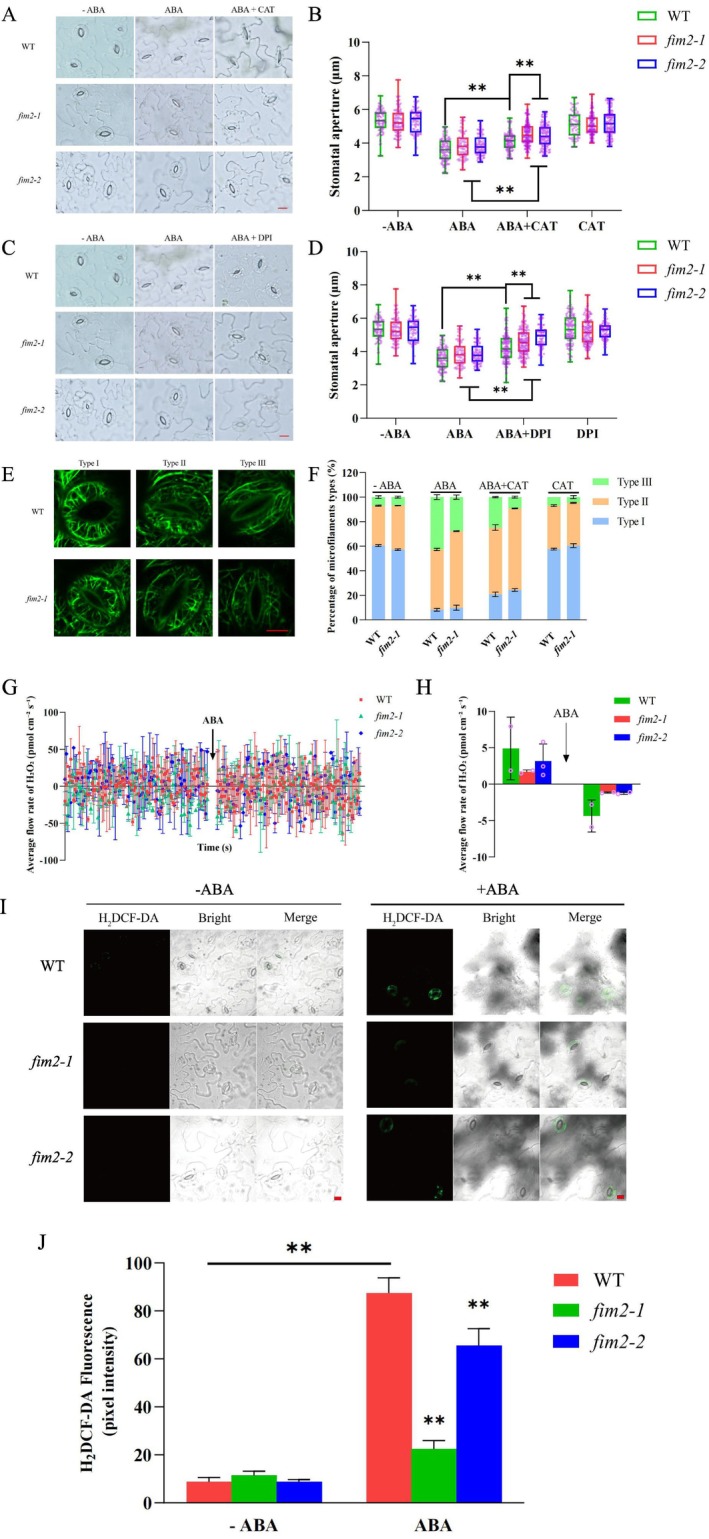
FIM2 affects ABA‐induced ROS dynamics in guard cells. (A) Representative images of stomatal aperture treated with ABA, CAT, or ABA + CAT. (B) Measurement of stomatal aperture treated with ABA, CAT, or ABA + CAT. (C) Picture of leaves treated with ABA, DPI, or ABA + DPI. (D) Measurement of stomatal aperture treated with ABA, DPI, or ABA + DPI. Each experiment was repeated three times independently as different biological replicates with 70 stomata of each seedling. **p < 0.01 (mean ± SD, two‐tailed *t*‐test), Bar = 10 μm. (E) Confocal microscopy images of microfilament arrangement in guard cells of WT and *fim2–1*, Bar = 5 μm. (F) Statistical data of WT and *fim2–1* on the proportion of microfilament arrangement treated with ABA, CAT, or ABA + CAT. (G) H_2_O_2_ flux in guard cells detected by noninvasive micro‐test technology (NMT). (H) Measurement of average H_2_O_2_ flux across the plasma membrane of guard cells. This experiment was repeated three times independently as different biological replicates. (I) Fluorescence images of H_2_O_2_ in guard cells before and after ABA treatment, Bar = 10 μm. (J) Fluorescence intensity of H_2_DCF‐DA in guard cells according to Figure 7I (at least 15 stomata were measured per group, mean ± SD, **p < 0.01).

First, we conducted pharmacological experiments to measure stomatal aperture. Catalase (CAT), a H_2_O_2_ scavenger, and diphenylene iodonium (DPI), an inhibitor of NADPH oxidase (a key generator of H_2_O_2_ in plant cells), were used to assess the potential role of endogenous H_2_O_2_ in FIM2‐involved stomatal movement. As shown in Figure 7A,B, exogenous application of ABA plus CAT significantly suppressed ABA‐induced stomatal closure in *fim2* mutants, rather than in the wild type (WT). A similar effect was observed with co‐treatment of ABA and DPI (Figure 7C,D). Both *fim2‐1* and *fim2‐2* lines exhibited more susceptible to ABA‐induced H_2_O_2_ accumulation than the WT, suggesting that H_2_O_2_ and FIM2 may cooperate during ABA‐induced stomatal movement.

Furthermore, we compared F‐actin reorganization in WT and *fim2* guard cells following treatment with ABA plus CAT. Confocal images were visually scored and categorized into three groups (Figure 7E,F). Co‐application of ABA and CAT largely impaired the characteristic longitudinal realignment of actin cables (Type III) observed in ABA‐induced closed stomata, due to the decreased cytosolic H_2_O_2_ levels scavenged by CAT. Moreover, the inhibitory effect of CAT was more pronounced in *fim2* mutants than in the WT, consistent with the stomatal aperture assays. These results suggest that both FIM2 and H_2_O_2_ contribute to the overall actin remodeling in response to ABA.

**FIGURE 8:**
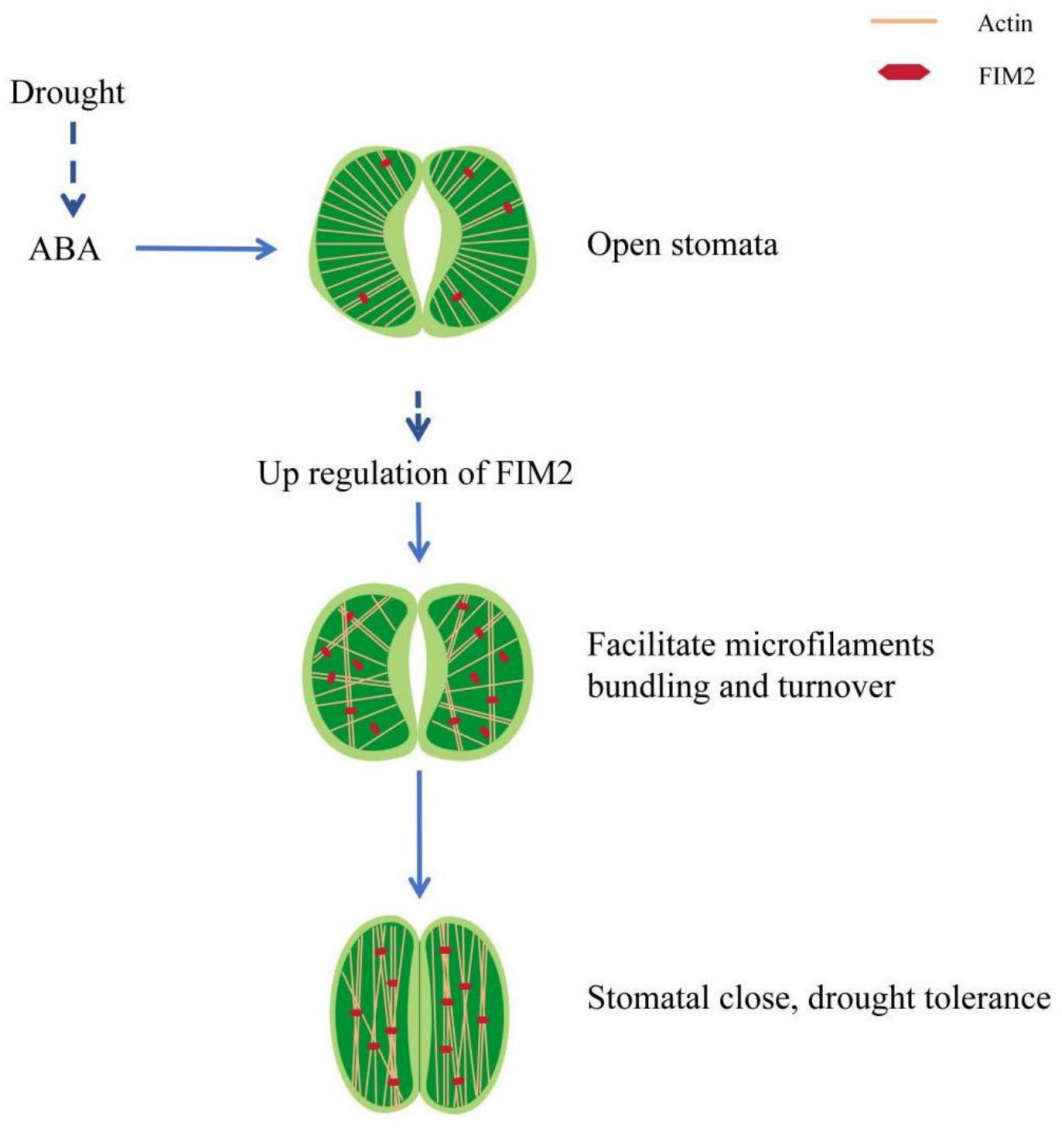
Proposed working model of FIM2 in regulating ABA‐induced microfilaments dynamics and stomatal closure in *Arabidopsis*.

Apoplastic H_2_O_2_ is primarily generated by NADPH oxidases on the plasma membrane, whose activity is influenced by actin dynamics (Liu et al. 2012). Fimbrin is a bona fide actin‐bundling protein that participates in actin dynamic process. Then we employed noninvasive micro‐test technology combined with H_2_DCF‐DA staining to monitor H_2_O_2_ changes in guard cells (Yang et al. 2023; Liu and Tominaga 2025). Upon treatment with 50‐μM ABA, the mean H_2_O_2_ influx into WT guard cells reached approximately 4.37 pmol·cm^−2^·s^−1^, significantly higher than that in the two *fim2* mutants (only 1.16 and 1.23 pmol·cm^−2^·s^−1^, respectively) (Figure 7G,H). In agreement with these observations, H_2_O_2_ accumulation detected by the specific fluorescent dye H_2_DCF‐DA was substantially attenuated by the mutation of FIM2, as indicated by weaker fluorescent signals in *fim2* guard cells (Figure 7I,J), indicating impaired ABA‐induced H_2_O_2_ production. These results suggest that ABA‐triggered H_2_O_2_ accumulation is compromised in *fim2* guard cells, leading to a diminished response to ABA signaling. Taken together, these findings suggest that FIM2 expression in guard cells is upregulated by ABA induction. FIM2 positively regulates ABA‐induced stomatal closure and inhibits transpirational water loss in leaves by modulating the dynamic turnover rate and bundling of actin filaments in guard cells, thereby affecting actin reorganization under ABA signaling (Figure 8).

## 3 Discussion

Stomatal closure is a core adaptive response for plants to cope with drought stress, and its precision depends on the integration of environmental signals, hormone transduction, and cytoskeletal remodeling (Li et al. 2022; Ren et al. 2024). In this study, we suggested FIM2 is participate in ABA signaling pathway, FIM2's expression is upregulated by ABA and mediate microfilaments rearrangement to facilitate stomatal closure and drought resistant in Arabidopsis. These findings not only clarified the functional role of FIM2 in stress responses but also deepen our understanding of how ABPs link hormone signals to cytoskeletal behavior in guard cells.

Drought stress triggers a series of physiological processes in plants, among which reducing transpirational water loss through stomatal closure is the most rapid and effective strategy (Lawson and Matthews 2020). Our phenotypic data show that FIM2 is involved in this process: Compared with WT plants, *fim2* mutants show more severe leaf wilting, higher water loss rates, and lower survival rates under drought conditions. The reduced leaf temperature of *fim2* mutants after ABA treatment further confirms that FIM2 deficiency leads to excessive water loss, which is consistent with the defective stomatal closure phenotype. These results collectively establish FIM2 as a positive regulator of drought tolerance. Notably, FIM2 expression is specifically upregulated by ABA in guard cells but not in mesophyll cells. Previous studies have reported that guard cells possess a unique signal transduction network to adapt to their functional specialization (Munemasa et al. 2015; Bharath et al. 2021). Our findings add a new component to this network: FIM2, as a guard cell‐enriched ABP, is partially required for ABA‐induced stomatal closing.

Actin filaments in guard cells undergo rapid and ordered rearrangement during stomatal closure: radial arrays (Type I) in open stomata disassemble into random meshworks (Type II) and further reorganize into longitudinal/fragmented arrays (Type III) in closed stomata (Shimono et al. 2016; Isner et al. 2017). This process is tightly regulated by multiple ABPs, and our study indicates FIM2 as a regulator in this coordination.

First, FIM2 is required for the efficient transition of actin filament patterns. In WT guard cells, ABA induces a sharp decrease in Type I and a significant increase in Type III within 30 min; in contrast, *fim2* mutants show a delayed transition, with most actin filaments remaining in the intermediate Type II. This block in pattern conversion directly explains the slower stomatal closure in mutants. Second, FIM2 enhances actin‐bundling capacity: Quantitative analysis shows that *fim2* mutants have reduced actin filament occupancy, lower skewness, and fewer detectable filament bundles in all pattern types. This is consistent with the conserved function of fimbrins as actin‐bundling proteins—FIM2 likely stabilizes actin filaments through its two ABDs; therefore, it plays a role in actin filament remodeling (Klein et al. 2004; Galkin et al. 2008).

A growing body of research indicates that microfilament remodeling is an important factor regulating the activity of ion channels and other membrane proteins. For example, actin dynamics have been shown to modulate voltage‐dependent calcium‐permeable channels in the guard cell plasma membrane of *Vicia faba* (Zhang and Fan 2009). Furthermore, depolymerization of F‐actin by Lat B enhances ABA‐induced H_2_O_2_ production by increasing the activity of the plasma membrane‐localized NADPH oxidase RbohD (Li et al. 2014). Both F‐actin and microtubules (MTs) participate in the dynamic distribution of the aquaporin AtPIP2;1 at the plasma membrane during flg22‐induced stomatal closure (Cui et al. 2021). Additionally, microfilament dynamics, along with several ABPs, such as the plant‐specific ABP SCAB1 and the ARP2/3 complex, is involved in regulating vacuolar morphology in guard cells (Yang et al. 2022; Li et al. 2013). The AP3 complex medium subunit AP3M cooperates with the actin cytoskeleton to mediate the transport of Golgi‐derived cargoes to the tonoplast, thereby promoting stomatal closure under drought stress (Zheng et al. 2019). More recently, the actin‐associated formin protein, rice morphology determinant (RMD) was shown to bind actin filaments and regulate stomatal opening in rice by modulating associations between the tonoplast and endoplasmic reticulum (Liu et al. 2025). Collectively, these findings demonstrate that dynamic reorganization of the actin cytoskeleton in guard cells, together with diverse ABPs, actively contributes to multiple working models for stomatal regulation. Unraveling the mechanistic roles of specific ABPs in stomatal movement, including FIM2, may be a promising direction for future studies.

Pharmacological experiments further support the dependence of FIM2 function on actin dynamics. Actin stabilizer Jas worsens the stomatal closure defect of *fim2* mutants, while the actin depolymerizer Lat B fails to promote closure in mutants as effectively as in WT plants. These results indicate that FIM2 is not only a structural regulator of actin filaments but also modulates their dynamic balance, which is essential for responding to ABA signals. In the root elongation zone of *A. thaliana* , both Lat B and jasmonate (JA) treatments reduce reactive oxygen species (ROS) levels. This reduction is absent in the atrbohC mutant, indicating that actin filament dynamics likely regulate ROS production through modulation of AtrbohC activity (Liu et al. 2012). Similarly, in root hair cells, actin filaments spatially regulate H_2_O_2_ accumulation by maintaining the plasma membrane localization of the NADPH oxidase RHD2 (Takeda et al. 2008). Parallel mechanisms exist in mammals, where H_2_O_2_ directly oxidizes specific cysteine residues (Cys‐139/Cys‐147) in cofilin to sulfinic acid, attenuating its actin‐binding and severing activity (Cameron et al. 2015). These observations support a model of reciprocal regulation between H_2_O_2_ and ABPs. On one hand, ABPs, by shaping actin filament dynamics, may influence the subcellular distribution and magnitude of H_2_O_2_ production in guard cells. On the other hand, H_2_O_2_ can post‐translationally modify ABPs, thereby altering their function and consequently remodeling the actin cytoskeleton. In this study, in the process of FIM2involved ABA signaling transduction, based on our results, we also propose that this bidirectional interaction forms a feedback loop that fine‐tunes the relationship between actin dynamics and H_2_O_2_. Such coordination is critical for the precise control of stomatal movements and contributes to adaptive responses under drought stress.

In addition, FRAP assays reveal that FIM2 promotes actin turnover: The fluorescence recovery rate of actin filaments in *fim2* guard cells is significantly lower than that in WT plants at both 60 and 420 s after photobleaching. This suggests that FIM2 is involved in maintaining actin dynamics—rapid turnover ensures that actin filaments can disassemble old structures (e.g., radial arrays) and assemble new ones (e.g., longitudinal arrays) in response to ABA, which is a key prerequisite for timely stomatal closure (Li et al. 2022).

The Arabidopsis genome contains five fimbrin genes (FIM1‐FIM5), which exhibit functional divergence. Previous studies have shown that FIM4 and FIM5 are mainly involved in root development and pollen tube growth by regulating actin organization (Wu et al. 2010; Zhang et al. 2016; Liu et al. 2024). However, the cellular function of FIM2 remains poorly characterized. Notably, a microarray analysis (Leonhardt et al. 2004) revealed that FIM2 belongs to a set of 64 transcripts detectable solely in guard cells, but not in mesophyll cells, in all three independent hybridizations. This preferential expression in guard cells strongly implies a specialized potential role of FIM2 in guard cell signaling. Given that ABA is a key phytohormone orchestrating stomatal movement to climate changes, the ABA‐induced upregulation of FIM2 in guard cells is physiologically coherent. Such upregulation likely contributes to the rapid reorganization of the actin cytoskeleton, which is essential for dynamic stomatal closure. Therefore, ABA‐triggered FIM2 expression may represent a regulatory mechanism ensuring swift and coordinated stomatal responses. This functional division within the fimbrin family reflects the evolutionary adaptation of plants: By assigning different paralogs to specific tissues (roots, pollen, and guard cells) and biological processes (development and stress), plants can efficiently coordinate actin‐dependent functions in response to internal and external cues.

Furthermore, although the dynamic remodeling of both microfilaments (F‐actin) and MTs during stomatal movement has been documented, along with numerous associated ABPs and microtubule‐associated proteins (MAPs) (Dou et al. 2021; Wang et al. 2023), the underlying molecular mechanisms are still not completely understood. Intriguingly, the spatial configuration of F‐actin and MTs in guard cells is somewhat similar in guard cells, suggesting their potential coordination in regulating stomatal movements. Some ABPs, such as formins, possess the ability to interact with both F‐actin and MTs (Wang et al. 2013; Sun et al. 2017), providing important clues for investigating the crosstalk and signal integration between these two cytoskeletal systems during stomatal movement. There is no doubt that in the near future, elucidating these mechanisms will eventually benefit plant resilience under the changing climate.

In summary, our study demonstrates that FIM2 enhances drought tolerance in Arabidopsis by mediating ABA‐induced stomatal closure. Its core function lies in regulating actin filament dynamics in guard cells: promoting actin bundling, facilitating ABA‐induced pattern rearrangement, and accelerating actin turnover to support rapid cytoskeletal remodeling. These findings reveal FIM2's role in ABA‐induced stomatal closure and have deepened our understanding of the role of the ABPs and actin cytoskeleton in plant stress adaptation.

## 4 Materials and Methods

### 4.1 Plant Materials and Growth Conditions

The Columbia (Col‐0) ecotype plants were used as WT. The T‐DNA insertion mutants of *fim2‐1* (SALK_026176) were kindly provided by Professor Shanjin Huang from Tsinghua University. The *fim2‐2* (SALK_063179) mutant was purchased by our laboratory from the NASC (Nottingham Arabidopsis Stock Centre) website. The GFP‐*f*ABD2 was generously donated by Professor Tonglin Mao from China Agricultural University and Professor Shanjin Huang from Tsinghua University.

After vernalization at 4°C for 3 days, the seeds were surface‐sterilized, then sown on 1/2 MS medium (2.2‐g·L^−1^ MS salts, 10‐g·L^−1^ sucrose, pH 5.6, 8‐g·L^−1^ agar) for 10 days of growth and transplanted into soil. The seedlings were grown under a 16‐h light/8‐h dark photoperiod in a growth room at 22°C.

### 4.2 Drought Treatment and Water Loss Assays

For the drought treatment, 14‐day‐old Arabidopsis seedlings were transplanted into pots with equal amounts of soil and initial water supply. They were then subjected to a 2‐week water deprivation period in a growth room under standard conditions, with a light period of 16 h of light and 8 h of darkness. In the water loss assay, rosette leaves were detached from 4‐week‐old plants and laid on a laboratory bench. The fresh weight of these detached leaves was recorded at 0.5‐h intervals over a total duration of 2 h. Water content was calculated and expressed as a percentage relative to the initial fresh weight.

### 4.3 Stomatal Aperture Measurement

Stomatal aperture assays were performed as described (Wang et al. 2023). *A. thaliana* plants grown for 3–4 weeks were selected, and leaves in good condition were excised and placed in MES buffer (50‐mmol·L^−1^ KCl, 10‐mmol·L^−1^ CaCl_2_, and 10‐mmol·L^−1^ MES, pH 6.15). The leaves were treated with a cold light source (light intensity: 500 μmol·m^−2^·s^−1^) at room temperature for 180 min to fully open the stomata, and then transferred to different treatment solutions (Figure S1A) for processing. The guard cells of the leaves were observed and photographed using a ZEISS Axiovert 200‐M inverted microscope, and the stomatal aperture was measured using ImageJ software.

### 4.4 Infrared Thermograph Imaging

Plants grown for 4 weeks were treated with 50‐μmol L^−1^ ABA for 3 h then photographed using a thermal imaging camera (VarioCAM HD), and leaf temperatures were measured with IRBIS3 Professional software. This method has been previously reported (Wang et al. 2023).

### 4.5 Laser Confocal Microscope Observation

Using *fim2‐1* plants expressing GFP‐*f*ABD2 (obtained through hybridization) and regular *GFP‐fABD2* plants as materials, leaves from 3‐ to 4‐week‐old Arabidopsis were irradiated with a cold light source in MES buffer to fully open the stomata. Then the leaves were subjected to 50‐μmol L^−1^ ABA in MES buffer for 30 min for ABA treatment. The lower epidermis of the leaves was peeled off to prepare slides, which were observed under a ZEISS 780 laser scanning confocal microscope (63× oil immersion objective, 488‐nm excitation light, zoom = 4). At least 40 cells were collected for each treatment, and the experiment was repeated three times. ImageJ software was used to analyze the actin cytoskeleton density, bundling degree, and angle, among other parameters.

### 4.6 Fluorescence Recovery After Photobleaching (FRAP)

FRAP of actin filaments in guard cells was performed using the FRAP function of the Leica SP8 X confocal microscope. The fluorescence recovery of actin filaments was observed, and the fluorescence recovery intensity of actin filaments was measured using LAS X software.

### 4.7 RNA Extraction and RT‐qPCR

Total RNA was extracted by using RNAprep pure Plant Kit (TIANGEN, #DP432). Then the RNA was subjected to reverse transcription with PrimeScript II 1st Strand cDNA Synthesis Kit (Takara, 6210A). RT‐qPCR was performed by using SYBR Premix Ex‐Taq (Takara, RR420A) in a CFX96 Real‐Time System (BIO‐RAD). The primers used were listed in Figure S1D.

### 4.8 Protoplast Preparation

To isolate guard cell protoplasts, rosette leaves from 3‐ to 4‐week‐old Arabidopsis plants were cut into thin strips. These strips were immersed in an enzyme solution (1.5% Cellulase R‐10, 0.4% Macerozyme R‐10, 0.1% BSA, 10‐mmol L^−1^ CaCl_2_, 20‐mmol L^−1^ KCl, 20‐mmol L^−1^ MES, 0.4‐mmol L^−1^ Mannitol, 50‐mmol L^−1^ β‐Mercaptoethanol, pH 5.5) and agitated at 26°C in the dark at 60 rpm for 2 h. The enzyme solution was then mixed with an equal volume of W5 (2‐mM MES, 0.9% NaCl, 125‐mmol L^−1^ CaCl_2_, 5‐mmol L^−1^ KCl, pH 5.7), filtered through 220‐ and 20‐μm filters, and the filtrate was centrifuged at 4000 rpm for 10 min. The supernatant was discarded, and this centrifugation step was repeated once. W5 was then added to the solution, which was incubated in an ice bath for 30 min. Guard cell protoplasts were collected by centrifugation at 1000 g for 5 min.

For mesophyll cell protoplast isolation, the epidermis was removed from rosette leaves of 3–4‐week‐old Arabidopsis plants using adhesive tapes. The remaining tissue was immersed in the same enzyme solution and agitated at 26°C in the dark at 60 rpm for 2 h. The enzyme solution was mixed with an equal volume of W5, filtered through a 220‐μm filter, and then processed using the same steps as for guard cell protoplast isolation.

### 4.9 Determination of H_2_O_2_ Flux in Guard Cells via Noninvasive Micro‐Test Technology

The real‐time flux rate of H_2_O_2_ across *A. thaliana* guard cells, namely, the H_2_O_2_ flux, was determined using the noninvasive micro‐test technology (NMT) system (NMT Physiolyzer, Younger USA LLC; Xuyue [Beijing] Sci. & Tech. Co. Ltd.). Epidermal peels were stripped from sample leaves, with guard cells distributed along the margin of the peeled (cut) area of the abaxial leaf surface. The peeled leaf segments were affixed to the bottom of a Petri dish using double‐sided adhesive tape, followed by immersion in the assay solution. After incubation for 60 min, the initial assay solution was discarded, and 5 mL of fresh assay solution was replenished prior to loading for measurement. Target guard cells were localized under a microscope, and the H_2_O_2_ flux sensor was positioned approximately 10 μm above the guard cells to initiate detection. After recording stable data for 10 min, the samples were treated with 50‐μM ABA for 15 min, followed by another 10 min of detection. Three biological replicates were performed for each experimental group. H_2_O_2_ flux data were directly retrieved using the imFluxes V2.0 software (YoungerUSA LLC, Amherst, MA 01002, USA). The flux was expressed in the unit of mol·cm^−2^·s^−1^, where positive values indicated H_2_O_2_ efflux and negative values represented H_2_O_2_ influx.

## Author Contributions

**Zixuan Wang:** investigation, writing. **Yuchong Han:** investigation. **Pan Wang:** investigation. **Pengfang Sun:** investigation. **Miao Hu:** investigation. **Rong Yu:** funding, conceptualization, data curation, supervision, project administration, writing.

## Funding

This research was supported by the National Natural Science Foundation of China (NFSC) (31871351).

## Conflicts of Interest

The authors declare no conflicts of interest.

## Peer Review

The peer review history for this article is available in the Supporting Information for this article.

## Supporting information


**Data S1:** Peer review.

**Table S1:** Solutions used in stomatal aperture measurement.
**Figure S1:** (A) Schematic diagram of T‐DNA insertion sites in *fim1* and *fim2* mutants. (B) Agarose gel electrophoresis of *fim2* mutants identified by three‐primer method (M = DNA Marker, WT = wild type). (C) The expression level of *fim2* mutants. Relative expression level was standardized with the reference *EF1‐α* gene, and three independent biological replicates were performed (two‐tailed *t*‐test, **p < 0.01).
**Table S2:** Primers used in this study.
**Figure S2:** Construction of the *proMYB60:FIM2* overexpression lines.

**Data S2:** Supporting information.

## Data Availability

All data are contained in this manuscript.
