# Supplementary material for: FIMBRIN2 Regulates ABA‐Induced Stomatal Closure by Promoting Microfilament Bundling and Turnover in Arabidopsis Guard Cells
Source: Plant Direct. 2026 Apr 12;10(4):e70162. doi: 10.1002/pld3.70162 (PMC13071139; doi:10.1002/pld3.70162)
Supplement: Supplementary file 2 — Table S1: Solutions used in stomatal aperture measurement. Figure S1: (A) Schematic diagram of T‐DNA insertion sites in fim1 and fim2 mutants. (B) Agarose gel electrophoresis of fim2 mutants identified by three‐primer method (M = DNA Marker, WT = wild type). (C) The expression level of fim2 mutants. Relative expression level was standardized with the reference EF1‐α gene, and three independent biological replicates were performed (two‐tailed t‐test, **p < 0.01). Table S2: Primers used in this study. Figure S2: Construction of the proMYB60:FIM2 overexpression lines. [file PLD3-10-e70162-s002.docx]

Supplementary Data

Table S1 Solutions used in Stomatal Aperture Measurement

| Solutions | Purpose |
| --- | --- |
| 50 μmol/L ABA | For ABA treatment |
| 50 μmol/L ABA+10 nmol/L LatB | For ABA+LatB treatment |
| 50 μmol/L ABA+20 nmol/L Jas | For ABA+Jas treatment |
| 50 μmol/L ABA+60 U CAT | For ABA+CAT treatment |
| 50 μmol/L ABA+10 μmol/L DPI | For ABA+DPI treatment |

Figure S1

A,

*
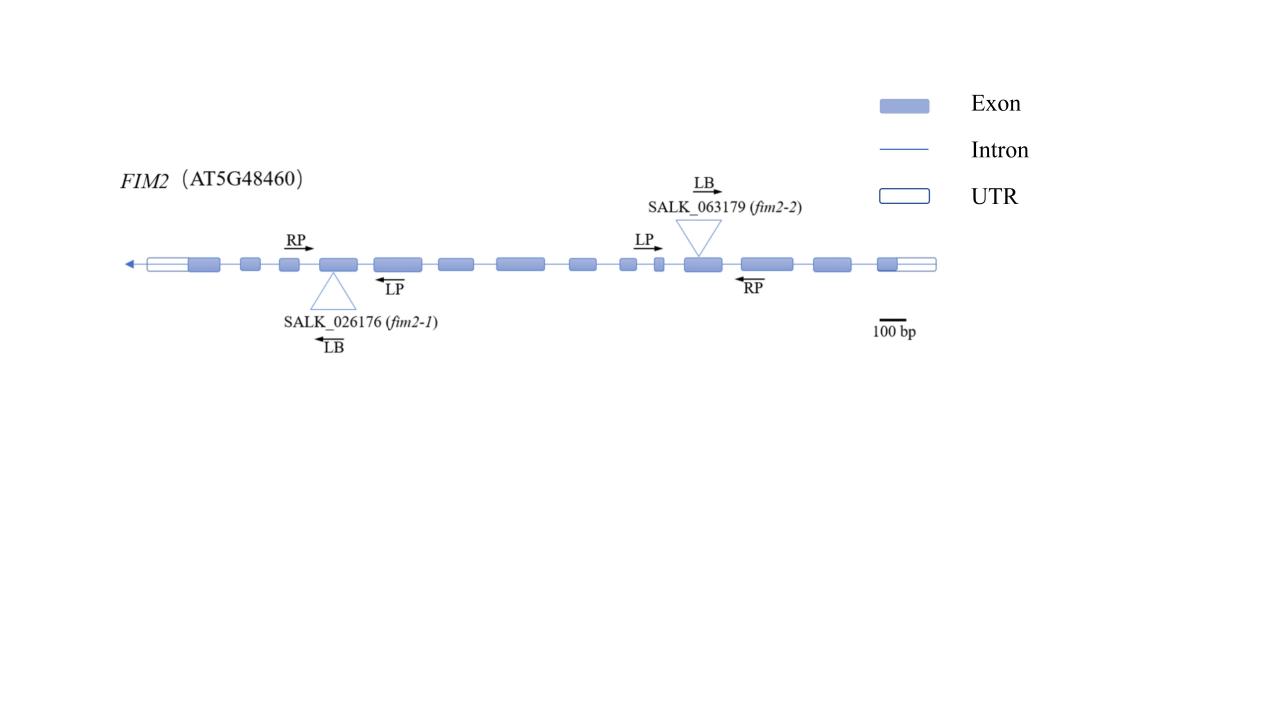
*

B


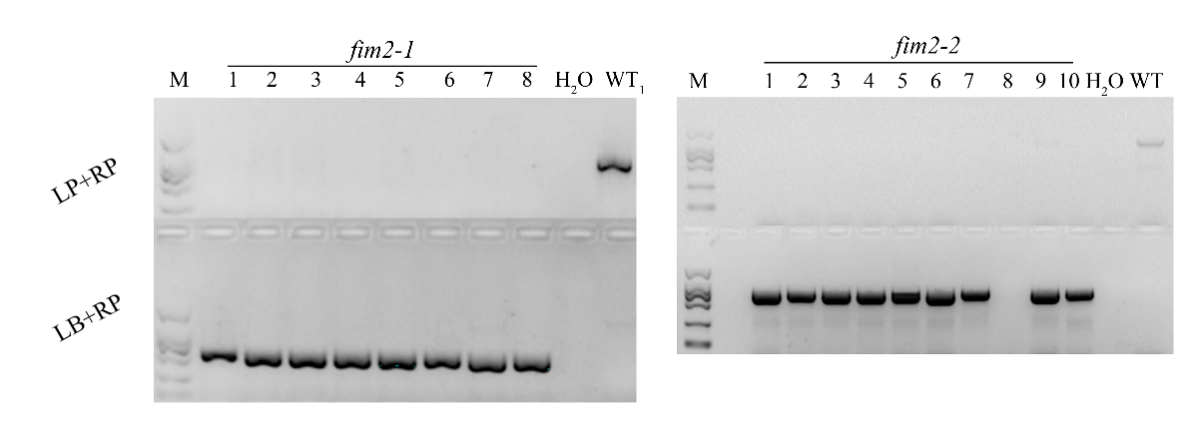


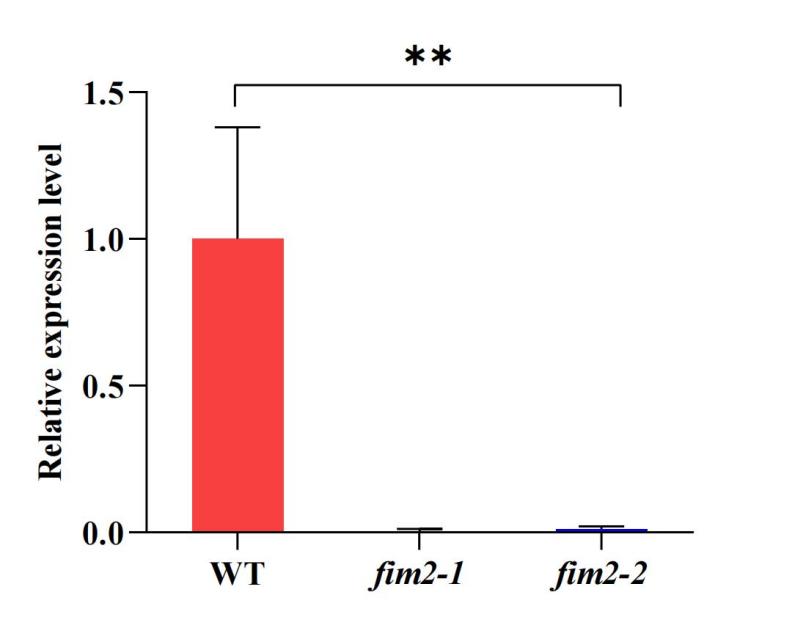
C

Figure S1 A, Schematic diagram of T-DNA insertion sites in fim1 and fim2 mutants. B, Agarose gel electrophoresis of fim2 mutants identified by three-primer method (M = DNA Marker, WT = wild type) C, The expression level of fim2 mutants. Relative expression level was standardized with the reference *EF1-α*gene, and three independent biological replicates were performed. (two-tailed t test, ***p<0.01*).

Table S2, Primers used in this study

| Primer | Sequence(5’-3’) | Purpose |
| --- | --- | --- |
| FIM2-1 LP | TTACCCGAGAGCTCATGTTTC | *fim2-1* Genotyping |
| FIM2-1 RP | TTCTCGATGTGATTGAGTGTTTG | *fim2-1* Genotyping |
| FIM2-2 LP | TGGGGATTGATTTTCTGTCTG | *fim2-2* Genotyping |
| FIM2-2 RP | AGGAACTGTCAATTGCCACTG | *fim2-2* Genotyping |
| LB 1 | ATTTTGCCGATTTCGGAAC | Genotyping |
| FIM2-QR | ATCCTTGCTATCACCGACCA | qPCR |
| FIM2-QF | CTATTGGGTGTACTGTGGTT | qPCR |
| FIM2-F | GAGAGAGAGAGAAAGCCATGGATGTCAGGATTCGTCGGGAT | Amplification of the FIM2 CDS fragment |
| FIM2-R | AAGTTCTTCTCCTTTACTAGTTTCTATAGAGGAATCTGACG | Amplification of the FIM2 CDS fragment |
| *proMYB60-F* | GACCTGCAGGCATGCAAGCTTCACAAGGACACAAGGACATATGG | Amplification of the MYB60 promoter fragment |
| *proMYB60-R* | ACTCTCAGGGTCCATAGATCTCTTTCTCTCTCTCTCTTCCTCTA | Amplification of the MYB60 promoter fragment |


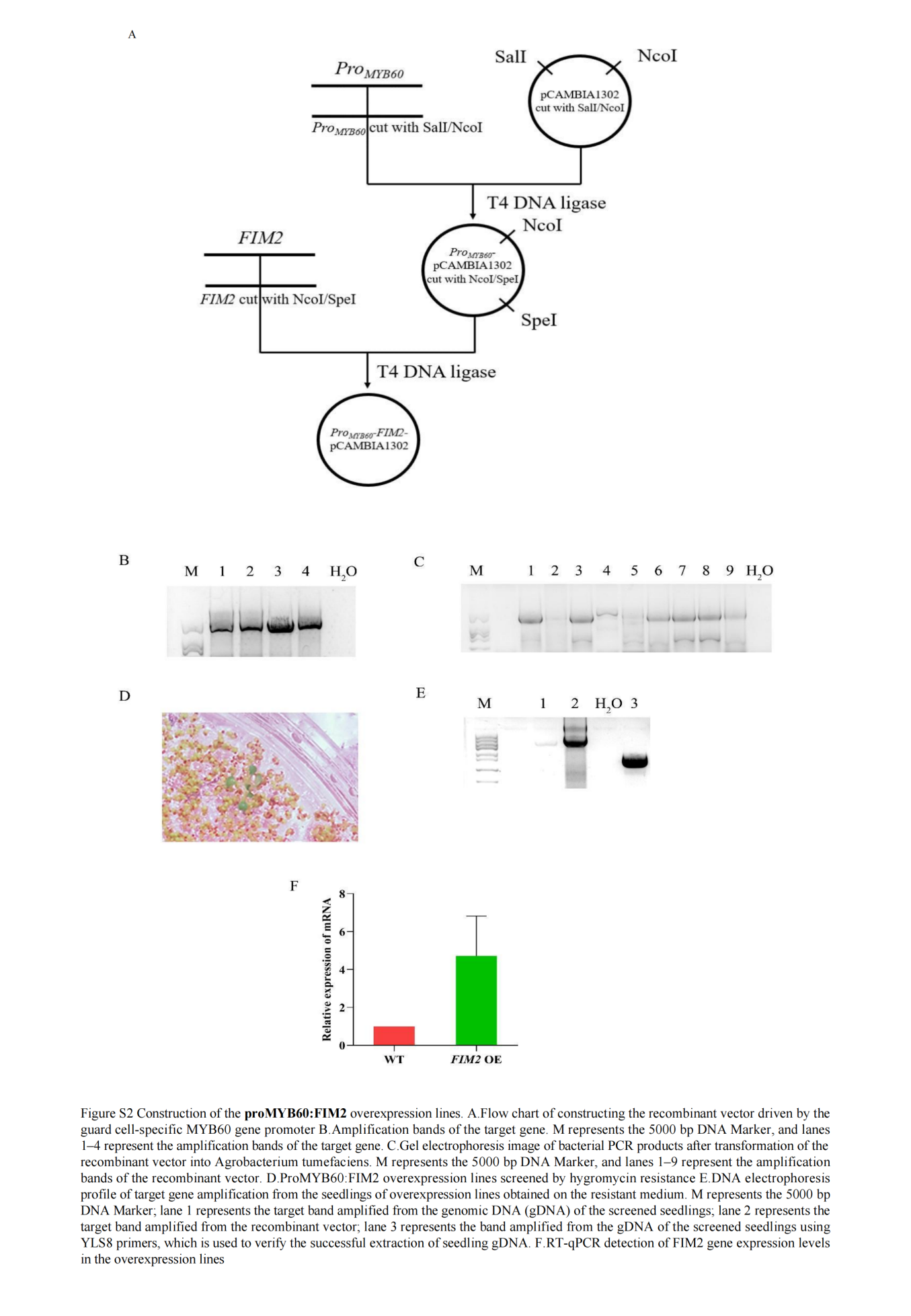
Figure S2 Construction of the *proMYB60:FIM2* overexpression lines.
