# Supplementary material for: FIMBRIN2 Regulates ABA‐Induced Stomatal Closure by Promoting Microfilament Bundling and Turnover in Arabidopsis Guard Cells
Source: Plant Direct. 2026 Apr 12;10(4):e70162. doi: 10.1002/pld3.70162 (PMC13071139; doi:10.1002/pld3.70162)
Supplement: Supplementary file 1 — Data S1: Peer review. [file PLD3-10-e70162-s003.pdf]

FIMBRIN2 Regulates ABA-Induced Stomatal Closure by Promoting Microfilament Bundling and Turnover in Arabidopsis Guard Cells.

Rong Yu, Zixuan Wang , Yuchong Han , Pan Wang , Pengfang Sun , Miao Hu

Decision letter Round 1:

November 15, 2025

Dr. Rong Yu

Capital Normal University

Beijing

China

RE: FIMBRIN2 Regulates ABA-Induced Stomatal Closure by Promoting Microfilament Bundling and Turnover in Arabidopsis Guard Cells.

Dear Dr. Yu:

Thank you for submitting to Plant Direct. All required reviews have been returned and we have now finished our evaluation of your manuscript. In light of the reviewers' and editor's comments, further revisions are needed before the paper can be accepted for publication in Plant Direct.

Please view the editors' and reviewers' comments below and use their suggestions as a guide while you work on your revision.

When uploading the revised version of this article, please be sure to include the following:

-A word document that contains your response to the reviewers. You should respond to each reviewer comment and note the changes made to the manuscript. If you do not agree with a reviewer's comment and choose not to make a suggested revision, please explain why. Please try to provide as complete an answer as possible to each reviewer's criticisms in the "response to reviewers" section.

-A tracked changes document

- A clean version of the latest version of the manuscript

To upload your revision, please click the link below.

https://plantdirect.msubmit.net/cgi-bin/main.plex?el=A1Lr7pv3A2otK1I3A9ftdXwvAIHz1OZG14cEisGGuFAZ

In order to provide as timely a service as possible, we ask that your revision is resubmitted within three months after receipt of this request. If an extension is needed, please send a request, along with a brief explanation, to the editorial office at plantdirect@wiley.com .

Thank you very much for giving us an opportunity to review your work. I look forward to receiving the next version.

Sincerely,

Marco Giovannetti

-- Reviewer comments:

Reviewer #1:

In the present manuscript by wang et al, the authors address an important question in guard-cell cytoskeletal regulation and provide valuable insight into how FIM2 modulates ABA-induced stomatal closure via effects on actin bundling and turnover. However, the confocal imaging and quantitative analyses are strong and technically impressive; I have a few queries and suggestions in the present format of the manuscript.

Though the fim2 mutant clearly displays delayed stomatal closure and altered actin dynamics, the inclusion of an overexpression and/or complementation line (e.g., native promoter-driven FIM2 in the fim2 background) would significantly strengthen the link between FIM2 disruption and the observed phenotypes. This would help exclude potential indirect effects of the mutation. Besides, the manuscript would benefit from further confirmation of FIM2 expression in guard cells. The use of a promoter::reporter line would help verify tissue specificity related to the observed phenotypes.

In Fig. 2b, the relative expression data show the calibrator sample with both a normalised mean of 1 and associated error bars. I believe that ΔΔCt values are calculated independently for each biological replicate; hence, some variation relative to the calibrator is expected, which should generate a non-zero SD. If the authors used only technical replicates for normalisation or averaged before normalisation, this could artificially reduce variance. The authors should clarify the replication structure (biological vs. technical replicates) and provide the exact normalisation steps used to generate the relative expression and error bars.

I agree that he stomatal aperture assay is informative; however, incorporating stomatal conductance or gas-exchange measurements would provide whole-plant physiological validation, connecting the cellular phenotype to drought sensitivity at the organismal level.

Furthermore, given the known interactions between actin organisation, $Ca^{2}+$ signalling, and ROS production during ABA responses, examining whether fim2 affects ABA-

induced Ca^{2}+ or ROS dynamics in guard cells could help position FIM2 more clearly within the ABA signalling pathway.

The manuscript would benefit from additional language polishing. In several parts, grammar and sentence structure could be improved to ensure the scientific message is conveyed clearly. I also recommend removing the periods at the end of subheadings, as this is not typical formatting in research papers and affects presentation quality.

Reviewer #2:

Wang et al. report that FIMBRIN2 is essential for ABA-induced stomatal closure by promoting actin microfilament bundling and turnover. An increasing body of research highlights the significance of microtubule rearrangements in stomatal closure. However, less is known about actin microfilaments, making this work nteresting to readers. Including a comparison of how actin and microtubules may work together, by clearly outlining this in the introduction and discussion, would be helpful.

1. The FIM gene family includes five paralogs. The authors should present GC expression profiles for all paralogs using publicly available data. Specifically, I am curious whether more than one paralog is expressed in guard cells. The authors might consider creating higher-order mutants, which could potentially show stronger phenotypes.

2. Since stomatal aperture depends on stomatal size and stomatal size varies greatly in Arabidopsis even within a single leaf, I strongly recommend reporting relative aperture by normalizing with stomatal height (width/height). There have recently been a number of non-reproducible studies measuring stomatal apertures in Arabidopsis. Conducting blind experiments can help reduce unintended bias, and blinding methods should be described in the methods (including how many people were involved in blinding and how)? This has become a standard in many laboratories and repeating these experiments as blinded is recommended for longer term impact of this study.

3. The authors should perform leaf gas exchange analysis to provide better evidence and time-course data on intact plants. Testing time-resolved stomatal conductance response to exogenous ABA using petiole feeding experiments is used by many labs, is strongly recommended and addresses the limitations of single stomatal aperture variance. The authors conclude on the rate of ABA responses and time-resolved gas exchange analysis will directly test this hypothesis.

4. Fig. 1: For the thermal imaging experiment, growing each genotype in separate pots may expose them to different levels of stress, which does not provide adequate experimental control. I strongly recommend growing multiple genotypes in a single pot, including genetic controls with contrasting leaf temperatures.

5. Line 27: Based on the presented findings, it appears that some conclusions may be overstated. The authors should rephrase the sentence to accurately reflect the result; FIM2 is partially required for ABA-induced stomatal closing.

Minor comments

6. Lines 15-20: Is microfilament reorganization enabling ion channels, membrane protein function, vacuole morphology, and vesicle trafficking true? Discussion addressing these points is recommended.

7. It is known that stomata close rapidly in response to exogenous ABA. Would the upregulation of FIM2 support such a rapid closing response? Or are there alternative reasons why FIM2 is genetically required for ABA-induced stomatal closure? The authors could further discuss this point.

8. It has been shown that strong signaling mutants, such as cop1 and ost1 mutants, exhibit impaired microtubule depolymerization, which the authors could discuss. Have the authors observed similar phenomena clearly for the actin structure in guard cells of a strong ABA signaling mutant? How might FIM2 and microfilament arrangement be regulated by the same upstream ABA signaling pathway?

9. Fig. 2: All bar graphs should display the raw data distribution, meaning individual measurements and Excel files with the data should be included, including for Figures 3B-F, since readers will be interested in the data from these, including new repeat experiments.

10. The authors could include images illustrating the microfilament structure after Jas, LatB, and ABA treatments to demonstrate that these pharmacological agents can effectively disrupt actin filaments in guard cells and relate to ABA-induced stomatal responses.

11. Fig. 4: A demonstrates different types of microfilaments. The authors should add a simplified cartoon to make it easier to understand.

Decision letter Round 2:

March 14, 2026

Dr. Rong Yu

Capital Normal University

Beijing

China

MSID: 2025-01852-TWR1

MS TITLE: FIMBRIN2 Regulates ABA-Induced Stomatal Closure by Promoting Microfilament Bundling and Turnover in Arabidopsis Guard Cells.

Dear Dr. Rong Yu:

I am pleased to inform you that your manuscript "FIMBRIN2 Regulates ABA-Induced Stomatal Closure by Promoting Microfilament Bundling and Turnover in Arabidopsis Guard Cells." has been accepted for publication in Plant Direct.

Your article will appear online in the next available issue of Plant Direct. To ensure your article gets published as quickly as possible, please pay attention to the steps detailed below. We have found that most of the delays happen at this stage, especially at the payment stage, so please respond as quickly as possible when prompted.

License Agreement: Once your article has been accepted it will move to Production and undergo admin and file checking - you may receive an email with any queries we have at this stage. When all required items are received by the publisher and queries resolved, the corresponding author will receive an email from Wiley's Author Services system which will ask them to log on at https://authorservices.wiley.com/bauthor and will present them with the appropriate license for completion. Your article cannot be published until both the signed license agreement and payment of the article fee have been received.

Payment of the Open Access Article Publication Fee: All articles published in Plant Direct are fully open access: immediately and freely available to read, download and share. Plant Direct charges a publication fee to cover the publication costs. The corresponding author for this manuscript should have already received a quote from the payments team (cs-openaccess@wiley.com) with the estimated article publication fee; please email cs-openaccess@wiley.com if this has not been received. The corresponding author should log on to the Wiley Author Services site, where the publication fee can be paid by credit card or an invoice. Pro Forma can also be requested. Payment of the publication charge must be received before the article will be published online.

Proofs: You will have the opportunity to look over your paper once more when you receive the author proofs for your article. The proofs will be with you in approximately two weeks. Please note that, in addition to publishing reviewer comments, the author's responses to review comments will also be published alongside the final version of the paper. If you would not like the author's responses to be published, please contact the editorial office at plantdirect@wiley.com .

Promotion of your article: You can help your research get the attention it deserves! Check out Wiley's free Promotion Guide for best-practice recommendations for promoting your work at www.wileyauthors.com/eeo/guide. And learn more about Wiley Editing Services which offers professional video, design, and writing services to create

shareable video abstracts, infographics, conference posters, lay summaries, and research news stories for your research at www.wileyauthors.com/eeo/promotion.

Thank you again for your contribution to Plant Direct. If you have any questions, feel free to contact the editorial office at plantdirect@wiley.com .

Sincerely,

Marco Giovannetti

Editor, Plant Direct

Response Letter Round 1:

Dear Editor,

We would like to express our sincere gratitude to you and the reviewers for the valuable

suggestions on our manuscript. We firmly believe that these suggestions will significantly improve

the quality of our work. We have revised our manuscript strictly according to the comments

outlined in the decision letter, and herein we provide responses to all the comments raised by the

reviewers in this response letter.

Reviewer #1:

1. Though the fim2 mutant clearly displays delayed stomatal closure and altered actin

dynamics, the inclusion of an over-expression and/or complementation line (e.g., native

promoter-driven FIM2 in the fim2 background) would significantly strengthen the link

between FIM2 disruption and the observed phenotypes. This would help exclude potential

indirect effects of the mutation. Besides, the manuscript would benefit from further

confirmation of FIM2 expression in guard cells. The use of a promoter::reporter line would

help verify tissue specificity related to the observed phenotypes.

Thanks very much for the thoughtful suggestions. As regarding the need for an

overexpression and/or complementation line and further confirmation of FIM2 expression in

guard cells to verify tissue specificity related to the observed phenotypes, we constructed guard

cell-specific MYB60 promoter to produce overexpression lines of FIM2 in the wild type

background ProMYB60:FIM2-OE (Supplemental Figure S2). Then the leaf water loss and stomatal

aperture measurements were performed (see Fig. 1C-D and Fig. 3A-B). The results are consistent

with their different FIM2 expression levels, which confirming the reliability of the plant materials

and strengthen the link between FIM2 disruption and the phenotypes. Furthermore, the use of the

MYB60 promoter provides supportive evidence for the guard cell-specific role of FIM2 relevant to

the phenotypic observations (Rusconi F et al., 2013; Zhang J et al., 2021;Nguyen CC et al., 2026).

However, due to the time-consuming nature of generating and fully characterizing stable

transgenic lines in plants, other OE and promoter:: reporter line related experiments are still

underway(Rusconi F et al., 2013).

References

1) Rusconi F, Simeoni F, Francia P, et al. The Arabidopsis thaliana MYB60 promoter provides a

tool for the spatio-temporal control of gene expression in stomatal guard cells. J.

Experimental Botany 2013, 64(11): 3361-3371

2) Zhang J, Zhou H, Zhou MJ, et al. The coordination of guard-cell autonomous ABA synthesis and DES1 function in situ regulates plant water deficit responses. J. Advanced Research 2021, 27:191-197

3) Nguyen CC, Thibivilliers S, Li YH, et al. Uncovering the core genetic programs governing plant guard cell biology. New Phytologist 2026, 249(1):198-217

2. In Fig. 2b, the relative expression data show the calibrator sample with both a normalised mean of 1 and associated error bars. I believe that ΔΔCt values are calculated independently for each biological replicate; hence, some variation relative to the calibrator is expected, which should generate a non-zero SD. If the authors used only technical replicates for normalisation or averaged before normalisation, this could artificially reduce variance. The authors should clarify the replication structure (biological vs. technical replicates) and provide the exact normalisation steps used to generate the relative expression and error bars.

We appreciate the reviewers' attention to the rigor of data analysis. In the statistical results presented in Figure 2B, guard cells showed a highly sensitive response to ABA signals. After treatment with ABA, the expression level of FIM2 in guard cells increased sharply, with the mean relative expression level rising from 1 before treatment to approximately 251-fold. Therefore, we adjusted the ordinate scale representing the relative gene expression levels when plotting the figure: the initial segment was scaled in units of 1, while within the range of 205–255, the same

length of scale represented 50 units. This compression of the numerical scale of the ordinate in the

high-expression interval led to the visual impression that the standard error of the mean (SEM) of

guard cells after ABA treatment was close to zero. In fact, the SEM was neither zero nor

artificially reduced, and the actual SEM value was 0.92. This visual illusion was entirely caused

by the scale compression of the ordinate in the high-expression interval.

In the RT-qPCR experiments, three independent extractions of protoplasts from guard cells

and mesophyll cells were performed, followed by RNA extraction and reverse transcription into

cDNA. For the qPCR assays, cDNA derived from the same batch of protoplast extraction was

applied to a single reaction plate, which was defined as one biological replicate. On each

individual reaction plate, three technical replicates were set up for the primer pairs amplifying

each gene, with the same cDNA sample added to each replicate.

The specific calculation steps for qPCR relative expression levels (the 2^{-}ΔΔCq method) were as

follows: first, the ΔCq value of each sample in both the control and experimental groups was

calculated by subtracting the Cq value of the reference gene(EF1-α) from that of the target gene;

second, the mean ΔCq value of the control group was calculated, and using this as the baseline, the

ΔΔCq value was obtained by subtracting the mean ΔCq value of the control group from the ΔCq

value of the experimental group; finally, the ΔΔCq value was substituted into the formula (2−ΔΔCq)

to calculate the relative expression level of the target gene in the experimental group compared

with the control group. A value greater than 1 indicates upregulated gene expression, whereas a

value less than 1 indicates downregulated gene expression.

The mean value of the relative expression levels from the three technical replicates within

each biological replicate was defined as the mean of that biological replicate, and the SEM value

was calculated using the relative expression levels of the three biological replicates.

3. I agree that he stomatal aperture assay is informative; however, incorporating stomatal

conductance or gas-exchange measurements would provide whole-plant physiological

validation, connecting the cellular phenotype to drought sensitivity at the organismal level.

Thanks a lot for the suggestion. Our aim is to elucidate the potential role of FIM2 in actin

filament reorganization during ABA-induced stomatal closure. In this context, stomatal aperture

assay emerges as an excellent method for our study, given its established and widespread use by

researchers in this field for evaluating plant tolerance or resilience against drought stress. To

further strengthen the physiological relevance of our findings, we also performed leaf water loss

assay and whole-plant drought stress treatment in soil. This experimental design follows a

logically progressive framework—from guard cells to leaf organs, and then to the whole plant.

And the results obtained are mutually consistent. Thus, our study effectively integrated the cellular

phenotypes with whole-plant physiological responses.

We acknowledge the reviewer's suggestion and concur that it would be both beneficial and

pertinent to better interpret the impacts of FIM2 on stomatal movement in future work by

incorporating stomatal conductance or gas-exchange measurements.

4. Furthermore, given the known interactions between actin organisation, Ca2+ signalling,

and ROS production during ABA responses, examining whether fim2 affects ABA-induced

Ca2+ or ROS dynamics in guard cells could help position FIM2 more clearly within the ABA

signalling pathway.

We thank the reviewer for this valuable suggestion. Following your advice, we thoroughly

review related references and focused on ROS, a critical signaling molecule in ABA signaling

pathway in response to drought stress for further investigation.

We subsequently performed a series of experiments, including pharmacological stomatal

aperture assay, confocal microscopy of actin cytoskeletal reorganization, and non-invasive

micro-test measurements of H_2O_2 fluxes under ABA treatment et al. The results, which we

consider encouraging, provide much better evidence to clarify the role of FIM2 in ABA-induced

stomatal closure. All relevant data have been incorporated into Figure 7 of the revised manuscript.

5. The manuscript would benefit from additional language polishing. In several parts,

grammar and sentence structure could be improved to ensure the scientific message is

conveyed clearly. I also recommend removing the periods at the end of subheadings, as this

is not typical formatting in research papers and affects presentation quality.

We appreciate the reviewer's suggestions on language and formatting. Accordingly, we have

carefully checked the manuscript sentence by sentence to refine the grammar and structure for

clearer scientific communication. The introduction section has been extensively revised and

expanded, providing a more detailed overview of the recent advances in the relevant research.

What’s more, the periods at the end of subheadings have been removed as recommended. And the

text has also been reviewed by a native English-speaking colleague to ensure clarity. We sincerely

hope the revised manuscript now meets the expected standards for presentation.

Reviewer #2:

Wang et al. report that FIMBRIN2 is essential for ABA-induced stomatal closure by

promoting actin microfilament bundling and turnover. An increasing body of research

highlights the significance of microtubule rearrangements in stomatal closure. However, less

is known about actin microfilaments, making this work interesting to readers. Including a

comparison of how actin and microtubules may work together, by clearly outlining this in

the introduction and discussion, would be helpful.

We appreciate the reviewer's suggestion. In response, we have expanded the Discussion

section to include an analysis of the potential coordination between microtubules and

microfilaments in guard cells during stomatal movement. Please refer to the revised Discussion for

details.(Revised manuscript, Page 13 line 25 to Page 14 line 7)

1.The FIM gene family includes five paralogs. The authors should present GC expression

profiles for all paralogs using publicly available data. Specifically, I am curious whether

more than one paralog is expressed in guard cells. The authors might consider creating

higher-order mutants, which could potentially show stronger phenotypes.

Thank you for this helpful suggestion. We have extensively searched the publicly available data and found that, in addition to FIM2, the presence of FIM1/4/5 in guard cells has also been reported (Nguyen C.C. et al., 2026 in supplements). However, functional studies on these iso-variants in guard cells remain limited.

Following the reviewer’s advice, we have begun constructing double and higher-order mutants (e.g., double and triple mutants) to further explore potential genetic interactions and functions in stomatal movement. Yet, given the three-month revision timeline and the need to perform other requested experiments (such as generating an overexpression line; positioning FIM2 within ABA-ROS pathway et al. by reviewer#1), this part of the work has not yet been completed.

We fully agree with the reviewer’s perspective that successful generation of FIM multiple mutants would indeed help us better elucidate the role of FIM family in stomatal signaling.

Reference:

Nguyen C.C., Thibivilliers S., Li Y., et al. 2026 Uncovering the core genetic programs governing plant guard cell biology. New Phytologist 249:198-217

2. Since stomatal aperture depends on stomatal size and stomatal size varies greatly in Arabidopsis even within a single leaf, I strongly recommend reporting relative aperture by normalizing with stomatal height (width/height). There have recently been a number of non-reproducible studies measuring stomatal apertures in Arabidopsis. Conducting blind experiments can help reduce unintended bias, and blinding methods should be described in the methods (including how many people were involved in blinding and how)? This has

become a standard in many laboratories and repeating these experiments as blinded is recommended for longer term impact of this study.

We appreciate the reviewer's valuable suggestion. We fully agree that the use of relative aperture can effectively reduce errors caused by variations in stomatal size. However, due to the large sample size involved in this study, which includes multiple replicate experiments, different drug treatments, and newly supplemented experimental data, all of these factors have taken up a substantial amount of time. Therefore, the re-measurement is still in progress, and we sincerely ask for your understanding.

Regarding the blind experiment, the blind method used for stomatal aperture data collection in this study is as follows: Researcher A labeled the experimental materials of each group and then handed them over to Researcher B for data collection. At this time, Researcher B was unaware of which experimental materials each label corresponded to. After data collection was completed, the data were submitted to Researcher C for processing. This process can reduce unintended bias from during data collection.

3.The authors should perform leaf gas exchange analysis to provide better evidence and time-course data on intact plants. Testing time-resolved stomatal conductance response to exogenous ABA using petiole feeding experiments is used by many labs, is strongly recommended and addresses the limitations of single stomatal aperture variance. The authors conclude on the rate of ABA responses and time-resolved gas exchange analysis will directly test this hypothesis.

We sincerely thank the reviewer for the constructive suggestion. Performing leaf gas

exchange analysis and petiole feeding assays to monitor time-resolved stomatal conductance in

response to ABA would indeed provide stronger direct evidence at the whole-plant level regarding

the role of FIM2 in ABA signaling kinetics. We fully agree that such experiments would further

support our conclusions from a complementary perspective.

In the present study, our experimental approach was designed following a multi-level

strategy—from the whole plant down to cellular responses—as commonly adopted in recent

high-impact studies (e.g., Dou et al. 2021, Fig. 3; Wang P. et al. 2023, Fig. 4; Zhong H. et al.

2024, Fig. 1). Specifically, we examined soil-drought phenotypes at the whole-plant level,

followed by leaf temperature and water loss assays at the organ level, and finally stomatal aperture

measurements in guard cells. This stepwise, integrative design ensures that findings are consistent

cross-validation and helps us address the inherent limitations of single stomatal aperture variance.

We appreciate the reviewer's emphasis on the importance of leaf gas exchange measurements,

time-resolved stomatal conductance et al. We are committed to incorporating leaf gas exchange

analysis, petiole-feeding experiments, and stomatal conductance assays in our follow-up work,

which we believe will substantially strengthen the mechanistic insights presented in this study.

Reference:

1) Dou L, He K, Peng J, et al. The E3 ligase MREL57 modulates microtubule stability and stomatal closure in

response to ABA. Nat Commun, 2021, 12: 2181

2) Wang P, Qi S, Wang X, et al. The OPEN STOMATA1–SPIRAL1 module regulates microtubule stability

during abscisic acid-induced stomatal closure in Arabidopsis. Plant Cell, 2023, 35: 260–278

3) Zhong H, Wang S, Huang Y, et al. Endomembrane trafficking driven by microtubule growth regulates

stomatal movement in Arabidopsis. Nat Commun, 2024, 15: 7967

Article

a Col-0 eb1a 1b 1c Light 120 min 60 min O min

COM-EB1a COM-EB1b b 4.5

https://doi.org/10.1038/s41467-024-52338-x

4.0

Col-0

☐ ebia 1b 1c

COMEB1a Stomatal aperture (um)

COM-EB16

a

a

a

b

3.0

b

b

d

d

2.5

cd d

b

C

2.0

1.5

1.0

0.5

C

1 56 0.0 n=128

n=159

n=101

=208

Col-0

0 min

n=154

n1177

125

0=215

ebia 1b 1c

60 min

n=213

n=146

=166

22.5

22.0-

d AtEB1b-mCherry

Light

120 min

GFP-TUA6

21.5-

Merge

19.6 22.2 (°C)

af temperatur 21.0-

20.5H

20.0

19.5-

19.0 n=269 n#282 Col-0 eb1a 1b 1c

Light Dark

4 Water loSS

(% of fresh weight)

100- +WT B -spr1-6 WT spr1-6 Com ost1-3

-ost1-3 80- -Com

SO-영

25

40-

20

0|

Temperature (°C)

a b

a

24 T

23

C

22

21

20

0| 1 2 3 4 5 6

WT

Time (h) D 5

a

C WT spr1-6 Com ost1-3

Control

Stomatal aperture (um)

a

a T

a b

b

spr1-6

Com

ost1-3

WT

spr1-6 T

d

C C

e Com

e ost1-3

د

Drought

1-ㄱ|

Rewatering O Control 1 10

ABA treatment (μM)

E F 60- ABA treatment

Control 1h 2 h

WT

spr1-6

Occupancy of MTs (%)

WT

a

a spr1-6

b

40- b

C d

20-

0|

Control 1 2

ABA treatment (h)

Figure 4 SPR1 positively regulates ABA-induced stomatal closure and drought avoidance. A, Fresh weights of detached leaves from different seedNATURE COMMUNICATIONS | https://doi.org/10.1038/s41467-021-22455-y

a -ABA +ABA e WT wd17-1 wd17-2 OE#1 OE#2

control

Relative expression

b 80-

60- control 1h a

0.5h 2h

drought

70/85 74/89 78/90 16/90 15/89

(82±3%) (83± 3%) (87 5%)X18 ± 9%)(17±3%) ** **

d

d d f 23 WT wd17-1 wd17-2 a

a a d d d d d

150 2 Relative expression of WDL7

WDL7 MREL57 RD29A 257

Temperature (°C) (9

b

مع

OE#1 OE#2

21 Wd17a17-ER wal O OE#2

g

d

wdl7-1 OE#1 OE#2

Water loss

(% of fresh weight)

30-

50-

O

WT

WT

--wd17-1

--wd17-2

40 OE#1

OE#2

20-

10

Stomatal aperture, um

WT □ wd17-1 wdl7-2 OEI OE/2

a

a a

T

C

a a C

e

T

C b b

e

e

2-

0

0| 0.5 1 1.5 2 2.5 3 0 control ABA ABA+MG132

ARTICL

Time, h

Fig. 3 Overexpression of WDL7 impairs the stomatal closure in response to ABA. a Detached rosette leaves from ProwDLXGUS transgenic seedlings we

incubated in opening buffer for 2 h and then treated with or without 10uM ABA for 2h The enidermal strins were peeled from the rosette leaves and Gl

4.Fig. 1: For the thermal imaging experiment, growing each genotype in separate pots may

expose them to different levels of stress, which does not provide adequate experimental

control. I strongly recommend growing multiple genotypes in a single pot, including genetic

controls with contrasting leaf temperatures.

We appreciate the reviewer’s valuable suggestion. Following your advice, we have sown

experimental materials with different genetic backgrounds simultaneously in the same culture dish.

The results are as follows: these findings are consistent with those presented in Figure 1E and

1F of the manuscript.

5. Line 27: Based on the presented findings, it appears that some conclusions may be

overstated. The authors should rephrase the sentence to accurately reflect the result; FIM2

is partially required for ABA-induced stomatal closing.

We sincerely thank the reviewer for the attentive and astute review of our work.As suggested,

we have revised the sentence to more accurately reflect the findings, clarifying that FIM2 is

partially required for ABA-induced stomatal closure.

Minor comments

6. Lines 15-20: Is microfilament reorganization enabling ion channels, membrane protein

function, vacuole morphology, and vesicle trafficking true? Discussion addressing these

points is recommended.

We are grateful to the reviewer's suggestion. In response, we have conducted a thorough

review of the reference and have incorporated a discussion of these key points into the revised

Discussion section. Details as follows：(Revised manuscript, Page 11 line 10 to Page 12 line 3)

"A growing body of research indicates that microfilament remodeling is an important factor

regulating the activity of ion channels and other membrane proteins. For example, actin dynamics

have been shown to modulate voltage-dependent calcium-permeable channels in the guard cell

plasma membrane of Vicia faba (Zhang and Fang, 2009). Furthermore, depolymerization of

F-actin by latrunculin B enhances ABA-induced H_2O_2 production by increasing the activity of the

plasma membrane-localized NADPH oxidase RbohD (Li et al., 2014). Both F-actin and

microtubules participate in the dynamic distribution of the aquaporin AtPIP2;1 at the plasma

membrane during flg22-induced stomatal closure (Cui et al., 2021). Additionally, microfilament

dynamics, along with several actin-binding proteins (ABPs), such as the plant-specific ABP

SCAB1 and the ARP2/3 complex, are involved in regulating vacuolar morphology in guard cells

(Yang et al., 2021; Li et al., 2013). The AP3 complex medium subunit AP3M cooperates with the

actin cytoskeleton to mediate the transport of Golgi-derived cargoes to the tonoplast, thereby

promoting stomatal closure under drought stress (Zheng et al., 2019). More recently, the

actin-associated formin protein RMD (Rice Morphology Determinant) was shown to bind actin

filaments and regulate stomatal opening in rice by modulating associations between the tonoplast

and endoplasmic reticulum (Liu et al., 2025). Collectively, these findings demonstrate that

dynamic reorganization of the actin cytoskeleton in guard cells, together with diverse ABPs,

actively contributes to multiple working models for stomatal regulation. Unraveling the

mechanistic roles of specific ABPs in stomatal movement, including FIM2, may be a promising

direction for future studies."

Reference:

1)Zhang W., and Fan L. M. (2009). Actin dynamics regulates voltage-dependent calcium-permeable channels of

the Vicia faba guard cell plasma membrane. J. Integr. Plant Biol. 51, 912–921

2)Li X., Li J. H., Wang W., Chen N. Z., Ma T. S., Xi Y. N., et al. (2014). ARP2/3 complex-mediated actin

dynamics is required for hydrogen peroxide-induced stomatal closure in Arabidopsis. Plant Cell Environ. 37,

1548–1560.

3)Cui Y., Zhao Y., Lu Y., Su X., Chen Y., Shen Y., et al. (2021). In vivo single-particle tracking of the aquaporin

AtPIP2;1 in stomata reveals cell type-specific dynamics. Plant Physiol. 185, 1666–1681. doi:

10.1093/plphys/kiab007

4)Yang Y., Zhao Y., Zheng W., Zhao Y., Zhao S., et al. (2022). Phosphatidylinositol 3-phosphate regulates

SCAB1-mediated F-actin reorganization during stomatal closure in Arabidopsis. Plant Cell 34, 477–494.

5)Li L. J., Ren F., Gao X. Q., Wei P. C., and Wang, X. C. (2013). The reorganization of actin filaments is required

for vacuolar fusion of guard cells during stomatal opening in Arabidopsis. Plant Cell Environ. 36, 484–497. doi:

10.1111/j.1365-3040.2012.02592.x

6)Zheng W., Jiang Y., Wang X., Huang S., Yuan M., and Guo Y. (2019). AP3M harbors actin filament binding

activity that is crucial for vacuole morphology and stomatal closure in Arabidopsis. Proc. Natl. Acad. Sci. U. S. A.

116, 18132–18141.

7)Liu Z.Y., Chang S.W., Yong M. et al. 2025 The formin protein RMD regulates rice stomatal opening by

modulating tonoplast-endoplasmic reticulum association. Cell Reports 44, 116689

7. It is known that stomata close rapidly in response to exogenous ABA. Would the

upregulation of FIM2 support such a rapid closing response? Or are there alternative

reasons why FIM2 is genetically required for ABA-induced stomatal closure? The authors

could further discuss this point.

We acknowledge the reviewer's suggestion and have accordingly addressed this point in the

Discussion section of the revised manuscript, as follows：(Revised manuscript, Page 13 line 7 to

line 24)

Among the five fimbrin isovariants in Arabidopsis, the cellular function of FIM2 remains

poorly characterized. Notably, a microarray analysis (Leonhardt N. et al., 2004) revealed that

FIM2 belongs to a set of 64 transcripts detectable solely in guard cells, but not in mesophyll cells,

in all three independent hybridizations. This preferential expression in guard cells strongly implies

a specialized potential role of FIM2 in guard cell signaling. Given that ABA is a key

phytohormone orchestrating stomatal movement to climate changes, the ABA-induced

upregulation of FIM2 in guard cells is physiologically coherent. Such upregulation likely

contributes to the rapid reorganization of the actin cytoskeleton, which is essential for dynamic

stomatal closure. Therefore, ABA-triggered FIM2 expression may represent a regulatory

mechanism ensuring swift and coordinated stomatal responses.

Reference:

Leonhardt N., Kwak J.M., Robert N., et al. 2004 Microarray expression analyses of Arabidopsis guard cells and

isolation of a recessive abscisic acid hypersensitive protein phosphatase 2C mutant. Plant Cell 16:596-615

8. It has been shown that strong signaling mutants, such as cop1 and ost1 mutants, exhibit

impaired microtubule depolymerization, which the authors could discuss. Have the authors

observed similar phenomena clearly for the actin structure in guard cells of a strong ABA

signaling mutant? How might FIM2 and microfilament arrangement be regulated by the

same upstream ABA signaling pathway?

We thank the reviewer for this suggestion.

In ABA signaling mutants, such as cop1, microtubule dynamics exhibit defects. Loss of

COP1 function results in the failure of normal microtubule depolymerization, resulting in the

persistent maintenance of their radial arrangement (Khanna R. et al. 2014; Chen Q. et al., 2021).

Similar to cop1 mutant, in the pyr1 pyl1 pyl2 pyl4 mutant, microfilament depolymerization is

significantly slowed down, and upon ABA treatment, the microfilaments in guard cells retain a

radial organization, losing the ability to form longitudinal bundles. PYR perceives ABA to form

the ABA-PYR-PP2C complex, which relieves PP2C-mediated inhibition of the CKL2 kinase.

Activated CKL2 phosphorylates actin-binding proteins such as ADF, thereby regulating the

dynamic remodeling of microfilaments and ABA-induced stomatal closure (Shi Y. et al., 2022).

Moreover, OST1/SnRK2.6, a central component of ABA signaling, interacts with MREL57

(MICROTUBULE-RELATED E3 LIGASE57), and the degradation of the microtubule-stabilizing

protein WDL7 is markedly delayed in ost1-3 in response to ABA treatment (Dou L. et al., 2021).

Given the similar dynamic patterns of microtubules and microfilaments during stomatal

movement, it is reasonable to speculate that the key ABA signaling components such as OST1,

CKL2 may also be involved in the FIM2-mediated stomatal closure process described in our study.

However, as we began procuring seeds for the cop1 and ost1 mutants only after receiving the

reviewer's comments, additional time is required for seed amplification and homozygous line

identification before experiments can be conducted. Therefore, we do not yet have conclusive

results to address this point. The experiments are currently underway, and we sincerely appreciate

the reviewer's patience and understanding.

Reference:

1)Khanna R., Li J., Tseng T-S., Schroeder J.I., Ehrhardt D.W., and Briggs W.R. 2014 COP1 jointly modulates

cytoskeletal processes and electrophysiological responses required for stomatal closure. Molecular Plant

7:1441-1454

2)Chen, Q., Bai, L., Wang, W., Shi, H., Ramón Botella, J., Zhan, Q., Liu, K., Yang, H. Q., & Song, C. P. (2021).

COP1 promotes ABA-induced stomatal closure by modulating the abundance of ABI/HAB and AHG3

phosphatases. The New phytologist, 229(4), 2035–2049. https://doi.org/10.1111/nph.17001

3)Shi, Y., Liu, X., Zhao, S., & Guo, Y. (2022). The PYR-PP2C-CKL2 module regulates ABA-mediated actin

reorganization during stomatal closure. The New phytologist, 233(5), 2168–2184.

https://doi.org/10.1111/nph.17933

4)Dou L., He K., Peng J. et al. 2021 The E3 ligase MREL57 modulates microtubule stability and stomatal closure

in response to ABA. Nature Communications 12:2181

9. Fig. 2: All bar graphs should display the raw data distribution, meaning individual

measurements and Excel files with the data should be included, including for Figures 3B-F,

since readers will be interested in the data from these, including new repeat experiments.

We appreciate the reviewer's valuable suggestions. In accordance with your advice, we have

added the original data distribution to Figure 2B. In addition to Figure 2, we have also

incorporated the measurement data from Figure 1B, Figures 5B–D, and Figures 6B–C into the

revised manuscript, and compiled all measured values into an Excel file, which is provided in

Supplementary Data Set 1 for your reference.

10. The authors could include images illustrating the microfilament structure after Jas, LatB,

and ABA treatments to demonstrate that these pharmacological agents can effectively

disrupt actin filaments in guard cells and relate to ABA-induced stomatal responses.

We thank the reviewer for the valuable suggestion to include images showing the effects of

Jas, Lat B, and ABA on actin filament organization in guard cells. In response, we would like to

clarify that Jasplakinolide (Jas) and Latrunculin B (Lat B) are well-established pharmacological

agents widely used in cytoskeleton research to specifically stabilize or depolymerize actin

filaments, respectively. Their well-characterized effects on actin dynamics have been consistently

demonstrated in numerous published studies (Kim et al., 1995; MacRobbie and Kurup, 2007; Li et

al., 2014; Zheng W. et al., 2019; Biel A. et al. 2020; Biel A. et al. 2022). For this reason, we relied

on these tools to find out the role of FIM2 in ABA-induced actin reorganization and stomatal

movement. Considering the focus and length constraints of the manuscript, we did not include

representative microscopy images of the drug-induced actin configurations, assuming their effects

were sufficiently recognized.

We fully acknowledge that incorporating such visual images would have provided clearer and

more direct support for our research, and we appreciate the reviewer’s reminder. In future studies,

we will make sure to systematically capture and retain such imaging data for a more

comprehensive presentation. Thank you again for your insightful comment and constructive

suggestion.

Reference

1)Kim, M., Hepler, P. K., Eun, S. O., Ha, K. S., and Lee, Y. (1995). Actin filaments in mature guard cells are

radially distributed and involved in stomatal movement. Plant Physiol. 109:1077–1084. doi:

10.1104/pp.109.3.1077

2)Li, X., Li, J. H., Wang, W., Chen, N. Z., Ma, T. S., Xi, Y. N., et al. (2014). ARP2/3 complex-mediated actin

dynamics is required for hydrogen peroxide-induced stomatal closure in Arabidopsis. Plant Cell Environ. 37,

1548–1560.

3)MacRobbie, E. A. C., and Kurup, S. (2007). Signaling mechanisms in the regulation of vacuolar ion release in

guard cells. New Phytol. 175: 630–640.

4)Zheng W., Jiang Y., Wang X., et al. (2019) AP3M harbors actin filament binding activity that is crucial for

vacuole morphology and stomatal closure in Arabidopsis. PNAS 116:36

5)Biel A., Moser M., and Meier I. (2020) A role for plant KASH proteins in regulation stomatal dynamics. Plant

Physiology 182:1100-1113

Biel A., Moser M., Groves NR and Meier I. (2022) Distinct roles for KASH proteins SINW1 and SINE2 in guard

cell actin reorganization, calcium oscillations, and vacuolar remodeling. Front. Plant Sci. 13:784342

11. Fig. 4 A demonstrates different types of microfilaments. The authors should add a simplified

cartoon to make it easier to understand.

We appreciate the reviewer's suggestion and have accordingly drawn a cartoon schematic to

illustrate the three distinct types of actin filament arrangement, as shown in the revised Figure 4A.
